# Anti-cancer drug sensitivity testing and preclinical evaluation of the anti-cancer potential of WEE1 inhibitor in triple-negative breast cancer patient-derived organoids and xenograft models

**DOI:** 10.1186/s13058-025-02063-0

**Published:** 2025-06-23

**Authors:** Seungyeon Ryu, Hoe Suk Kim, Sangeun Lee, So-Hyun Yoon, Moonjou Baek, A Young Park, Han-Byoel Lee, Ga Yeon Kim, Kyung Hyeun Park, Ji-Jung Jung, Yireh Han, Dong Woo Lee, Bosung Ku, Wonshik Han

**Affiliations:** 1 Department of Surgery, Seoul National University College of Medicine, Seoul, Republic of Korea; 2 Cancer Research Institute, Seoul National University, Seoul, Republic of Korea; 3 Biomedical Research Institute, Seoul National University Hospital, Seoul, Republic of Korea; 4 Interdisciplinary Programs in Cancer Biology Major, Seoul National University Graduate School, Seoul, Republic of Korea; 5 Integrated Major in Innovative Medical Science, Seoul National University Graduate School, Seoul, Republic of Korea; 6 Genomic Medicine Institute, Medical Research Center, Seoul National University, Seoul, Republic of Korea; 7 Department of Biomedical Engineering, Gachon University, Seongnam, 13120 Republic of Korea; 8 Central R&D Center, Medical & Bio Decision (MBD) Co., Ltd, Suwon, 1629 Republic of Korea

**Keywords:** Patient-derived organoid (PDO), Patient-derived xenograft (PDX), PDX-derived organoid (PDXO), High-throughput screening (HTS), WEE1, AZD1775

## Abstract

**Background:**

Relevant surrogates that maintain the pathological and physiological properties of patient tumors are essential for guiding triple-negative breast cancer (TNBC) therapy. The goals are to generate patient-derived organoids (PDOs), xenografts (PDXs), and PDX-derived organoids (PDXOs), evaluate the therapeutic potential of the WEE1 inhibitor AZD1775, and compare their responses to 18 anti-cancer drugs in PDOs and PDXOs.

**Methods:**

PDOs were produced from surgical specimens of patients with TNBC. PDXs were generated by transplanting PDOs into the mammary fat pads of NOD.Cg-Prkdcscid Il2rgtm1wjl/SzJ mice. PDXOs were derived from fresh tumor specimens of PDXs. For drug efficacy, half-maximal inhibitory concentration (IC50) values for 18 anti-cancer drugs on PDOs and PDXOs were calculated using the CellTiter-Glo® 3D cell viability assay in a high-throughput drug screening system. The relationship between WEE1 expression and survival in TNBC-basal-like (BL) patients was analyzed using the Kaplan–Meier Plotter database. Mice were treated with AZD1775 via oral gavage (30 mg/kg). Biological mechanisms underlying the anti-cancer drug responses were evaluated by calcein-AM staining, caspase 3/7 staining, Western blot, flow cytometry, and immunohistochemistry.

**Results:**

PDOs were established through subcultures of 2-7 passages. TNBC-BL PDXs expressing CK5, vimentin, and EGFR were generated and expanded over 3-4 generations of transplantation. PDXOs were produced through subcultures of 4-5 passages. PDOs, PDXs, and PDXOs retained the immunohistological characteristics of the relevant patients with TNBC. WEE1 was associated with poor survival outcomes in TNBC-BL patients. The highest cytotoxicity and tumor growth suppression to AZD1775 therapy were observed in PDXOs and PDXs with high WEE1 expression. AZD1775 inhibited WEE1 and CDK1 phosphorylation, increased γH2AX phosphorylation, induced G2/M arrest, and activated caspase 3/7 in PDXOs and PDXs, all associated with DNA damage, mitotic catastrophe, and apoptosis. Anti-cancer drug responses were highly concordant between matched PDOs and PDXOs. The responses of PDOs and PDXOs to anti-cancer drugs were comparable to those of patients receiving neoadjuvant or adjuvant chemotherapy, according to clinical records.

**Conclusion:**

PDOs, PDXOs, and PDXs, which maintained the immunological properties of TNBC patient, provide a scientific rationale for future WEE1-targeted clinical trials in TNBC. PDOs and PDXOs represent cost- and time-effective surrogates for predicting prioritized personalized therapy.

**Supplementary Information:**

The online version contains supplementary material available at 10.1186/s13058-025-02063-0.

## Background

Triple-negative breast cancer (TNBC) is characterized by the absence of estrogen receptors (ER), progesterone receptors (PR), and human epidermal growth factor receptor 2 (HER2) amplification, accounting for 10–15% of all breast cancers [1]. TNBC is an aggressive and heterogeneous disease classified into six molecular subtypes: basal-like (BL1 and BL2), mesenchymal (M), mesenchymal stem-like (MSL), immunomodulatory (IM), and luminal androgen receptor (LAR) [2]. It can also be categorized into four tumor-specific subtypes: BL1, BL2, M, and LAR [3]. A significant proportion of TNBC consists of the BL subtype, which expresses both cytokeratin 5/6 (CK5/6) and/or epidermal growth factor receptor (EGFR) [4, 5]. Due to the lack of well-defined molecular targets, TNBC poses challenges for effective targeted therapy. Consequently, there remains an unmet clinical need to identify more precise druggable targets for patients with TNBC. Furthermore, a relevant preclinical model for anti-cancer treatment is essential to address the challenges posed by the aggressiveness and heterogeneity of TNBC.

Patient-derived tumor organoids (PDOs) and patient-derived tumor xenografts (PDXs) have been recognized as relevant models in both fundamental research and precision medicine due to their ability to replicate the pathological and physiological characteristics of patient tumors [6–9]. PDOs enable high-throughput screening (HTS) and demonstrate concordant drug responses, making them optimal preclinical models compared to PDX models with limitations such as long experimental periods, high costs, and inability to access HTS. Thus far, PDO models have been established for many human cancers, including pancreatic [10], lung [11], liver [12], and breast cancer [8, 9]. However, the limited amount of starting patient tissue restricts the application of extensive pharmacological approaches. In contrast, large collections of PDX-derived organoids (PDXOs), pathophysiologically equivalent to PDXs, have proven feasible for drug screening and discovery [13]. PDXOs may overcome the limitations of PDOs and PDXs and are efficiently utilized for selecting sensitive anti-cancer drugs as cost-effective and time-saving surrogates for the personalized treatment of patients with TNBC [13]. However, as studies investigating whether the anti-cancer drug responses of TNBC-PDXOs mirror those of matched TNBC-PDOs and -PDXs are still lacking, further research is needed.

WEE1 is the primary gatekeeper for G2/M phase checkpoints and is crucial in regulating the cell cycle and repairing DNA damage [14, 15]. Elevated WEE1 levels are associated with aggressiveness in diverse human cancers including glioma [16], melanoma [17], and breast cancer [18]. Recently, WEE1 has been highly expressed in TNBC patients who exhibit the BL1 subtype [18]. Additionally, the heightened sensitivity of TNBC cell lines to WEE1 inhibitor and the independent poor prognostic significance of WEE1 expression in patients with TNBC samples have been documented [18], suggesting that WEE1 is emerging as a potential biomarker for prognosis and a therapeutic target. However, in a Phase II clinical trial involving patients with metastatic TNBC, the combination of a WEE1 inhibitor and cisplatin resulted in an objective response rate (ORR) of 26%, falling short of the pre-specified ORR cutoff of 30% [19]. The clinical trial did not account for WEE1 protein levels in the TNBC patient cohorts, and few studies have explored the relationship between WEE1 protein expression and the WEE1 inhibitor. Therefore, we conducted drug screening with a WEE1 inhibitor using PDO, PDXO, and PDX models to determine whether WEE1 protein may serve as a predictive biomarker.

We generated PDOs, PDXs, and PDXOs that preserved the major immunohistochemical characteristics of TNBC patients. To evaluate PDOs and PDXOs as patient surrogates for clinical drug testing, we investigated the efficacy of various anti-cancer drugs in PDOs and PDXOs, comparing them to follow-up records of patients receiving neoadjuvant (NCT) or adjuvant (CTx) chemotherapy. We also assessed the therapeutic potential of the WEE1 inhibitor (AZD1775) in matched PDOs, PDXOs, and PDXs to provide the scientific rationale for future WEE1-target-driven clinical trials in TNBC patients.

## Methods

### Human specimen

Breast tumor tissues were obtained from patients operated at the Seoul National University Hospital (Seoul, Republic of Korea). This study was approved by the Institutional Review Board of Seoul National University Hospital (approval number: H-2007–204–1145). The experiments were undertaken with the understanding and written consent of each subject. This study was performed in accordance with the Declaration of Helsinki.

### Anti-cancer drugs

Doxorubicin (brand name Adriamycin), Paclitaxel (brand name Abraxane), Docetaxel, Capecitabine (brand name Xeloda), Gemcitabine, Palbociclib (CDK4/6 inhibitor), Lapatinib (EGFR inhibitor), Olaparib (PARP inhibitor), Taselisib (PI3KCA inhibitor), Everolimus (mTOR inhibitor), AZD6738 (ATR inhibitor), AZD1775 (WEE1 inhibitor), Quisinostat (HDAC inhibitor), XAV-939 (Tankyrase inhibitor), and Wnt-C59 (WNT inhibitor) were purchased from Sellekchem (Houston, Texas, USA). Eribulin, Carboplatin, and Cisplatin were purchased from AdooQ Bioscience (Irvine, CA, USA). Anti-drug drug details are provided in Table 1.

**Table 1:** The detail information of anti-cancer drugs

NO	Drug	Brand	Cat. No	Stock Con. (µM)	Maximum Con. (µM)	Dilution ratio	Solvent	Formulation	Storage
1	Doxorubicin	Selleckchem	S1208	0.6	3	3	DMSO	Liquid	−80C in solvent
2	Paclitaxel	AdooQ	A10689	40	200	3	DMSO	Liquid	
3	Docetaxel	AdooQ	A10326	40	200	3	DMSO	Liquid	
4	Eribulin	AdooQ	A12804	0.6	3	3	DMSO	Liquid	
5	Capecitabine	Selleckchem	S1156	40	200	3	DMSO	Liquid	
6	Gemcitabine	Selleckchem	S1714	50	250	3	DMSO	Liquid	
7	Carboplatin	AdooQ	A10182	20	100	3	Water (warmed)	Liquid	
8	Cisplatin	AdooQ	A10221	20	100	3	DMSO	Liquid	
9	Palbociclib	Selleckchem	S1116	20	100	3	DMSO	Liquid	
10	Lapatinib	Selleckchem	S2111	10	50	3	DMSO	Liquid	
11	Olaparib	Selleckchem	S1060	10	50	3	DMSO	Liquid	
12	Taselisib	Selleckchem	S7103	0.6	3	3	DMSO	Liquid	
13	Everolimus	Selleckchem	S1120	10	50	3	DMSO	Liquid	
14	AZD6738	Selleckchem	S7693	2	10	3	DMSO	Liquid	
15	AZD1775	Selleckchem	S1525	2	10	3	DMSO	Liquid	
16	Quisinostat	Selleckchem	S1096	0.6	3	3	DMSO	Liquid	
17	XAV-939	Selleckchem	S1180	0.6	3	3	DMSO	Liquid	
18	Wnt-C59	Selleckchem	S7037	0.6	3	3	DMSO	Liquid	

### Organoid culture

Tumor tissues were processed as described previously with minor amendments to establish PDOs and PDXOs [8]. In brief, fresh surgical tissues of five TNBC patients and three PDX lines were cut into 1–3 mm^3^ pieces. Small fragments were digested in basal media containing advanced DMEM/F12 (Invitrogen, Carlsbad, CA, USA) with a mixture of collagenase/hyaluronidase (Stemcell Technologies, Vancouver, British Columbia, Canada) and DNase I (Roche, Basel, Basel-Stadt, Switzerland) in shaker incubator at 37℃ for 3 h. The digested tissue suspension was strained over a 100 µm filter and centrifuged at 500 g for 5 min. The pellet was resuspended in advanced DMEM/F12 with 10 mM HEPES, and 1% penicillin/streptomycin, centrifuged again at 500 g twice, and re-suspended with a Matrigel (Corning, NY, State of New York, USA) at density of 2000 cells per l.5 µl. The Matrigel dome was overlaid with full organoid media reported in a previous study and incubated at 37℃.

PDOs (*n* = 5) and PDXOs (*n* = 3) were propagated following the previously described protocol with minor modifications [7]. The organoids were passaged every 10 days using Matrigel recovery solution (Corning). The organoids above 100 µm in diameter were broken down into smaller clusters of cells by TrypLE™ (Thermo Fischer, Waltham, MA, USA) and re-embedded in Matrigel domes as described above.

### High-throughput screening (HTS)

For HTS and implementation of three-dimensional (3D) cell culture models, 1.5 µl of mixtures containing approximately 4000 cells and 50% Matrigel were automatically dispensed onto the 96-pillar plate surface using a solenoid valve (The Lee Company, Westbrook, CT, USA) and ASFA™ Spotter ST (Medical & Bio Decision, Suwon, Republic of Korea). Each plate comprised 96 wells, which contained 200 µl of cell culture medium. Cells were stabilized in the Matrigel spots for 2–3 days before being treated with drugs. After six days of treatment with the anti-cancer drugs, the drug response was analyzed through viability quantification of 3D live-cell images of calcein- acetoxymethyl-ester (calcein-AM) staining (Invitrogen). Green fluorescence intensities (excitation/emission, 494/517 nm to lasers) on the 96-pillar surface were measured using an automatic optical fluorescence scanner (ASFA™ Scanner HE, Medical & Bio Decitwesion) and an 8-bit code among the RGB codes (0–255). The total and average green areas of the scanned images of cultivated cells in each 3D cell culture model were calculated using the ASFA Ez SW (Medical & Bio Device). To determine therapeutic efficacy, dose–response curves were plotted using normalized 3D cell viability values corresponding to drug dose. The half-maximal inhibitory concentration (IC_50_) values were automatically determined during the XY analysis using the GraphPad software (GraphPad Prism 8, GraphPad Software, San Diego, CA, USA). Table 1 summarizes the drug concentrations exposed to PDOs and PDXOs.

### Cell viability and apoptosis

3D cell viability was assessed using Calcein-AM staining (Invitrogen) and Celltiter-glo® 3D Cell Viability Assay Kit (Promega, Chilworth, Southampton, UK,) according to the manufacturer’s protocol. To determine intracellular esterase activity, the staining solution was prepared with 4 mM of Calcein-AM in a culture medium. Pillar plates were incubated for 30 min at 37℃ and 5% CO_2_ incubator, then scanned by ASFA™ Scanner (Medical & Bio Decision). Following the image scanning, the Celltiter-glo® 3D Cell Viability Assay Kit was used to determine cell viability in 3D cell culture based on the quantification of intracellular ATP levels. In brief, the working solution was mixed at a 1:1 ratio with culture medium and CellTiter-glo® 3D reagent. After mixing, 40 µl of the working solution was added to 384 opaque white well plates. Pillar plates were incubated at room temperature for 30 min in the dark. Luminescence was measured by TECAN spark Cyto (Tecan, Männedorf, Zürich, Switzerland).

Caspase 3/7 activities in live cells were detected using CellEvent™ Caspase 3/7 Green Detection Reagent (Thermo Fischer). After the drugs were treated, 1 drop/ml CellEvent Caspase 3/7 green detection reagent was added to the 384 well plate and incubated for 72 h. The intensity of the green fluorescence and bright field images were visualized through ASFA™ Scanner (Medical & Bio Decision). The images of green fluorescence were analyzed using Image J software (National Institutes of Health, Bethesda, MD, USA).

### Western blotting

PDO, PDXO, and tumor tissues were lysed in RIPA buffer mixed with protease and phosphatase inhibitor cocktail (Thermo Scientific). Protein samples (20–40 µg) were separated on 8 or 15% SDS-PAGE and then transferred to a polyvinylidene difluoride membrane (Millipore). After blocking with 5% BSA in TBS-T at room temperature for 1 h, membranes were subjected to incubation with primary antibodies, including β-actin (Sc-47778, C4, 1:2000, Santa Cruz Biotechnology, Dallas, TX, USA), WEE1 (Sc-5285, B-11, 1:200, Santa Cruz Biotechnology), phospho-CDK1 (Tyr15) (#9111, polyclonal, 1:1000, Cell Signaling Technology, Beverley, MA, USA), phospho-WEE1 (Ser642) (#4910, D47G5(B7), 1:1000, Cell Signaling Technology), and phosphor-histone γH2AX antibody (05–636, JBW301, 1:1000, Millipore, Billerica, MA, USA), overnight at 4℃. Secondary antibodies were Horseradish Peroxidase (HRP)-conjugated goat-anti-rabbit IgG (#7074, polyclonal, 1:2000, Cell Signaling Technology) and HRP-conjugated goat-anti-mouse-IgGκ (Sc-516102, Recombinant, 1:2000, Santa Cruz Biotechnology), both at a dilution of 1:2000. These were incubated at room temperature for 1 h, followed by visualization using SuperSignal West Femto Chemiluminescent Substrate (Thermo Scientific) and Amersham™ ECL™ Prime Western Blotting detection reagent (GE Healthcare Life Sciences, Nightingales Ln, Chalfont, UK). The relative intensities of the bands observed by Western blotting were analyzed using Image J software (National Institutes of Health).

### Immunohistochemistry (IHC)

The primary tumors and lung were fixed with 4% buffered paraformaldehyde, embedded in paraffin blocks, and sectioned into 4 µm thick sections. The sections were deparaffinized at 60℃ for 1 h, and twice in xylene for 10 min, rehydrated in a series of graded ethanol (100%, 95%, and 70%) and water solutions, and pretreated at 98℃ for 20 min in citrate (pH 6.0) or 10 mM Tris/1 mM EDTA (pH 9.0) solution for antigen retrieval. Endogenous peroxidase activity was blocked by peroxidase inhibition buffer (Dako) at room temperature for 20 min. After incubation with blocking solution (0.05 mol/L Tris–HCl buffer containing 0.1% Tween; Dako) for 1 h to block nonspecific binding of immunological reagents, each primary antibody was incubated at 4℃ overnight. Primary antibodies used were; ER-alpha (NCL-L-ER-6F11, 6F11, 1:50, Novocastra, Newcastle upon Tyne, Tyne and Wear, UK) and PR (NCL-L-PGR-312, Clone 16, 1:100, Novocastra), HER-2/neu (790–4493, 4B5, ready to use, Ventana Medical Systems, Bas-Rhin, Illkirch, France), Ki67 (M7240, MIB-1, 1:100, Dako, Glostrup, Denmark), WEE1 (Sc-5285, B-11, 1:50, Santa Cruz Biotechnology), Ki67 (Sc-23900, L428, 1:100, Santa Cruz Biotechnology), and Vimentin (Sc-6260, V9, 1:200, Santa Cruz Biotechnology), Cytokeratin 5 (ab75869, EPR1600Y, 1:100, Abcam, Trumpington, Cambridge, UK) and EGFR (ab52894, EP38Y, 1:200, Abcam), phospho-CDK1 (Tyr15) (#9111, polyclonal, 1:100, Cell Signaling Technology), phospho-WEE1 (Ser642) (#4910, D47G5(B7), 1:100, Cell Signaling Technology), and phosphor-histone γH2AX antibody (05–636, JBW301, 1:200, Millipore). Then, slides were incubated with HRP-conjugated secondary antibody (Goat anti-rabbit/mouse Envision System HRP, K5007, polyclonal, ready to use, DAKO) for 30 min, and immunoreaction was visualized using the DAB chromogen kit (Agilent Technologies, Produktionsvej, Glostrup, Denmark). Nuclei were counterstained with hematoxylin solution (Millipore) according to the manufacturer's instructions. Histological images of the stained tissues were acquired using a microscope equipped with a CCD camera (Leica, Wetzlar, Hessen, Germany).

### Cell cycle analysis

PDXOs cultivated as two-dimensional (2D) monolayers in a collagen type I coated plate with full organoid media. When 2D monolayers cells grew at 70% confluence, cells were treated with DMSO (maximum 0.2%) and AZD1775 to 1, 2, 4 µM for 24 h. Then, cells were fixed in 70% cold ethanol overnight at 4℃. Subsequently, 10 µg/ml propidium iodide (Sigma-Aldrich, Burlington, MA, USA) and RNase A (2 mg/ml) were added, and cell cycle analysis was performed using flow cytometry (BD Bioscience). Data were analyzed using ModFit 3.0 (BD Bioscience).

### DNA damage detection

To detect DNA damage marker, p-γH2AX (Ser139), PDX-derived cells were treated with AZD1775 for 48 h, fixed in 70% cold ethanol at 4℃, and incubated with the antibody at its recommended concentration for 20 min at room temperature. For staining the nucleus, 4’,6-diamidino-2-phenylindole (DAPI) (1 µg/ml) was allowed to react for 15 min. Subsequently, samples were analyzed on a BD Calibur flow cytometer (BD Bioscience).

### PDX models and administration of AZD1775 in vivo

NOD.Cg-Prkdc^scid^ Il2rg^tm1wjl^/SzJ mice (NSG mice) were obtained from The Jackson Laboratory (Bar Harbor, ME, USA). Animal care and experimental procedures were conducted following the animal’s ethical guidelines. The study protocol was approved by the Institutional Animal Care and Use Committee of Seoul National University (SNU-150210–3–4). All methods were reported following ARRIVE guidelines (https://arriveguidelines.org) for the reporting of animal experiments. Five-week-old female NSG mice were injected into the fourth mammary fat pad with PDOs suspended in 1X PBS and Matrigel (Corning) at a 1:1 ratio for each mouse (exception for PDX#1; injection with spheroids, due to failure to organoid harvest). Serial transplantation was performed three times after tumors reached approximately 800 mm3, though the PDX tumor tissues chopped into 2-3mm^3^ were orthotopically engrafted into NSG mice.

For metastasis observation, following the surgical resection of primary PDX tumors, the mice had been housed for a further 2–3 months and were sacrificed. Major organs including lung and liver were collected and H&E staining and immunohistochemical analysis of CK5 were performed.

For in vivo drug test, vehicle (0.5% methylcellulose; *n* = 5) or AZD1775 (30 mg/kg; *n* = 5) was administered through oral gavage for 3–4 weeks with 5 days on and 2 days off. Tumor volumes were measured using a digital caliper twice per week and a modified ellipsoidal formula (Volume = 1/2(length × width^2^)), was employed for calculations.

### Survival analysis

The relationship of WEE1 genes with overall survival (OS) of TNBC-BL patients (*n* = 158), with a mean follow-up of 120 months was evaluated by the Kaplan–Meier (KM) Plotter surveyed public microarray data repositories. The cutoff value of WEE1 expression was chosen as the median, which split the patient samples into two groups, and plots were generated accordingly. ER, PR, and HER2 status was determined by both IHC and prediction analysis of microarray 50 (PAM50).

### Statistics

Data were presented as the mean ± standard deviation of at least three independent experiments. Statistical analyses were performed using GraphPad Prism v9.2.0 (GraphPad Software). Statistical comparisons between the two groups were conducted using the unpaired t-tests. The statistics of groups of three or more were evaluated using one-way ANOVA followed by Tukey's multiple comparison test. For all tests, a *p*-value < 0.05 was considered statistically significant. Statistical significance was defined as *p < 0.05, **p < 0.01, ***p < 0.001.

## Results

### PDOs recapitulated the immunohistological characteristics of the original tumor tissues of TNBC patients

For PDO cultures, we obtained tumor tissue from five TNBC patients who underwent total mastectomy with informed consent. The detailed clinical and pathological characteristics of the TNBC patients are presented in Table 2 and Fig. 8. Patient#5 was diagnosed with metaplastic breast cancer, while the others had invasive ductal carcinoma. Cytokeratin 5/6 (CK5/6)-positive cancer cells were found in patients #1, #2, #3, and #5. High Ki67 expression was noted in patient#1 (60%), patient#2 (70%), and patient#3 (35%). Additionally, Patient#3, initially diagnosed with metastatic breast cancer, had lesions in both the lung and liver.

**Table 2:** Clinical parameters of breast cancer patients

Case No	Age	Intrinsic subtype	Dx	Tumor Size (cm)	Pathology stage	Metastatic site	Surgical specimen IHC							
							**NG**	**HG**	**ER** **(%)**	**PR** **(%)**	**HER2**	**AR** **(%)**	**Ki67** **(%)**	**CK5/6** **(±)**
#1	40	TNBC	IDC	2.5	T3N1M0		3	3	< 1	0	-	5	60	+
#2	70	TNBC	IDC	3.7	T2N0M0		3	3	0	0	-	0	70	+
#3	45	TNBC	IDC	4.1	T3N1M1	lung, liver, hilar LNs	3	3	0	0	-	0	35	+
#4	57	TNBC	IDC	1.4	T1N1M0		3	2	0	0	-	0	1	-
#5	51	TNBC	Metaplastic	18	T4N1M0		3	3	0	0	-	1	20	+

*IHC* immunohistochemistry, *Dx* disease type, *IDC* invasive ductal carcinoma, *NG* nuclear grade, *HG* histologic grade, *ER* estrogen receptor, *PR* progesterone receptor, *HER2* human epithelial receptor 2, *AR* androgen receptor, CK5/6 cytokeratin 5/6, *+* positive, — negative

**Fig. 1:**
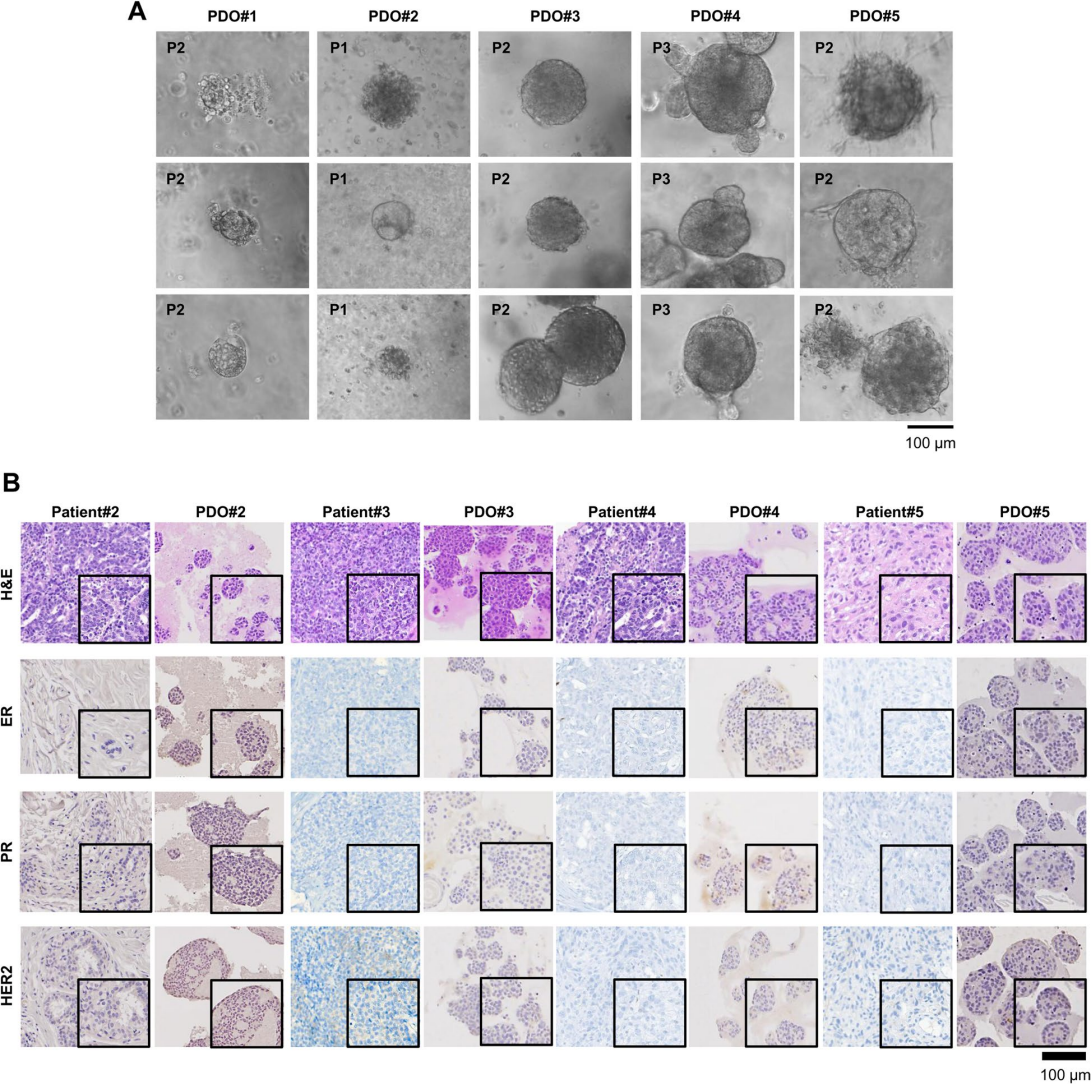
PDOs from surgical tumor tissues of TNBC patients recapitulate the immunohistological characteristics of the original tissues. **A** Microscopy images depicting major morphological phenotypes of PDO. **B** Representative images of H&E staining and immunohistochemistry of ER, PR, and HER2 in the surgical specimens of patients and PDOs. PDO, patient-derived organoid; H&E, hematoxylin & eosin; ER, estrogen receptor; PR, progesterone receptor; HER2, human epithelial receptor 2

We successfully established PDOs #2, #3, #4, and #5 through three to four subcultures, but we could not expand PDO#1. PDOs #2, #3, #4, and #5 grew as cohesive and solid spheres of varying sizes, as observed by bright-field, while PDO#1 exhibited grape-like and round shapes (Fig. 1a). PDOs matched the patient’s tumors in terms of ER, PR, and HER2 status as determined by immunohistochemistry (Fig. 1b).

### PDXOs and PDX tumors preserved immunobiological characteristics comparable to TNBC patients with the BL subtype

**Fig. 2:**
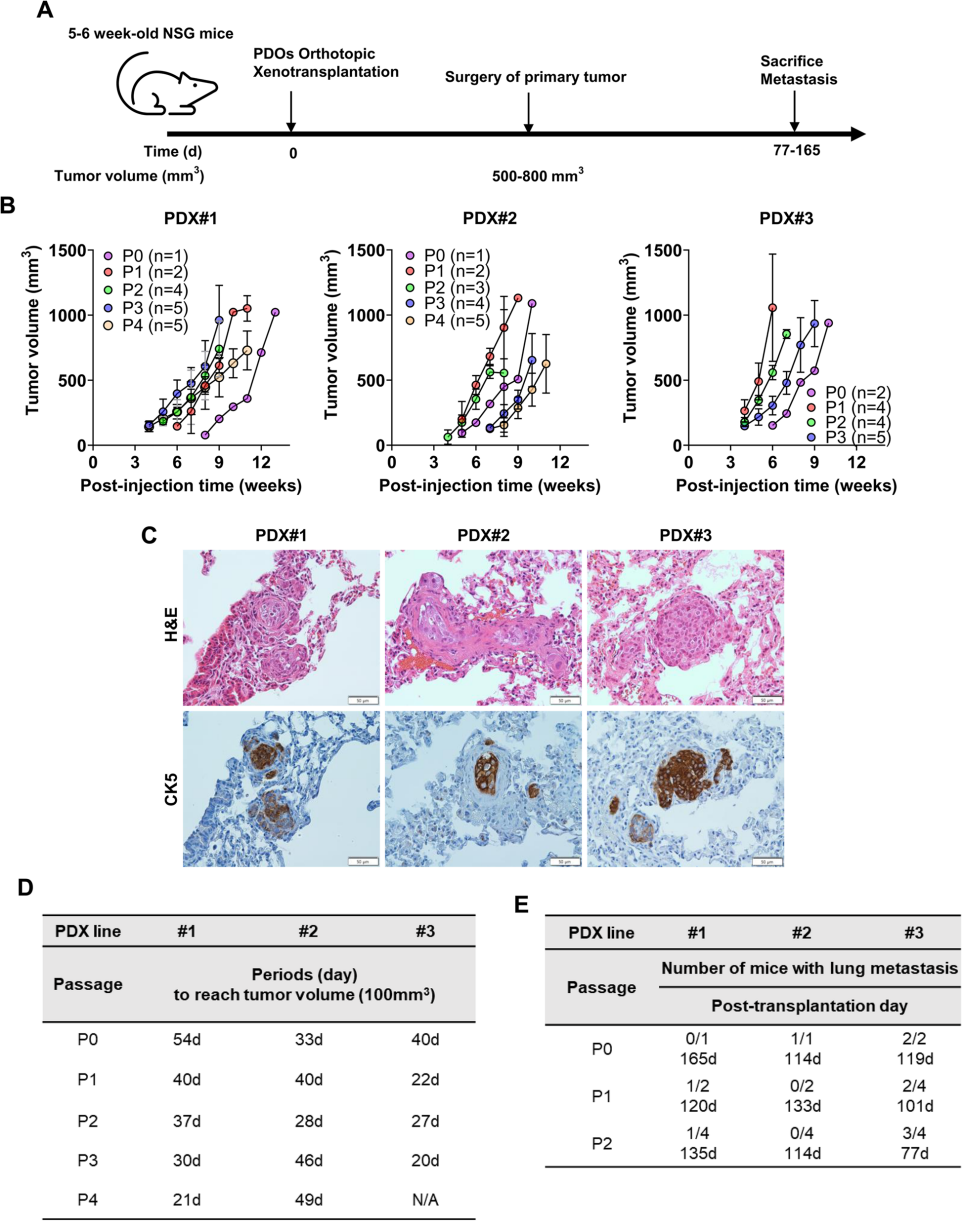
Validation of PDX model transplanted by PDOs and comparison of lung metastasis frequency. **A** Experimental design and timeline. **B** Tumor volume was measured with a digital caliper weekly in PDX mice from P0 to P4. **C** Representative H&E and IHC images of CK5 in lung tissue of PDX mice. **D** The period to reach 100 mm.^3^ of tumor volumes in PDX mice from P0 to P4. **E** The number of PDX mice with lung metastasis and periods to detect lung metastasis by CK5 immunostaining in PDX mice from P0 to P2. PDX, patient-derived xenograft; CK5, cytokeratin 5

To generate the PDX model, PDO#2, #3, #4, and #5, which have diameters over 100 µm, or 3D multicellular spheroid#1 derived from patient#1, were injected into the mammary fat pad of NSG mice (Fig. 2a). After removing the primary tumor, when it was 500–800 mm^3^, PDX mice were sacrificed on post-transplantation days 77–165 (Fig. 2a). We successfully generated PDX mice from PDO#2, PDO #3, and spheroid#1 within 1–2 months but failed to generate PDX mice from PDO#4 and PDO#5 after six months. PDX#1, #2, and #3 mice were maintained through serial transplantation of fresh tumor specimens for up to three or four generations. The tumor volumes were measured every 7 days after transplantation. Compared to PDX#1 and #2, PDX#3 developed tumors more rapidly (Fig. 2b). Lung metastases, not liver metastases, were detected via CK5 immunostaining in PDX#1, #2, and #3 (Fig. 2c). The time required to reach a 100 mm^3^ tumor volume in the P1- P4 generations was shorter than the P0 generation (Fig. 2d). The P1 and P2 generations required less time to develop lung metastases than P0 generation mice (Fig. 2e). Compared to PDX#1 and #2, PDX#3 mice exhibited fast-growing tumors and lung metastases, similar to the metastatic progression observed in the lung, liver, and hilar lymph nodes diagnosed in patient#3.

**Fig. 3:**
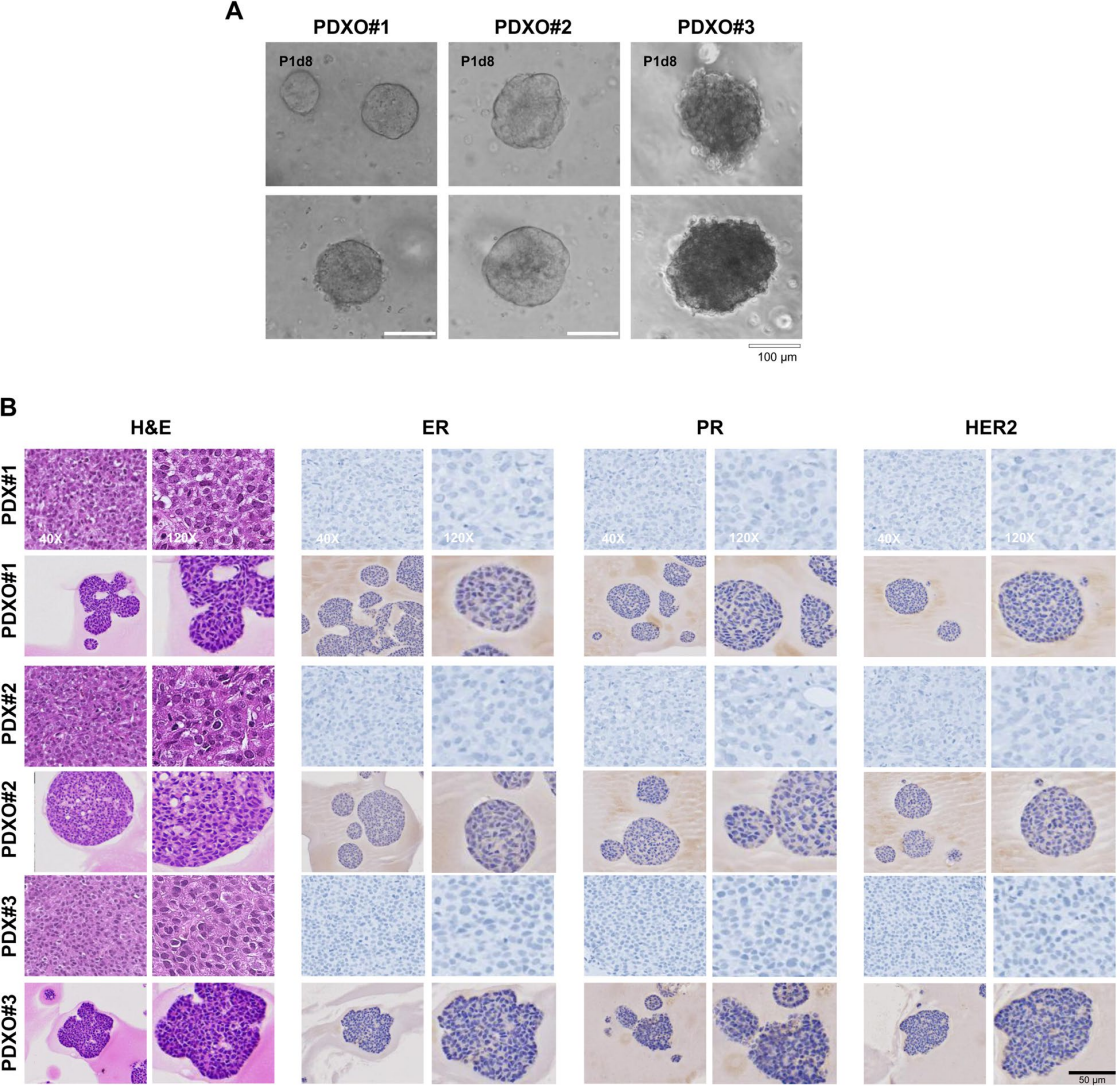
PDX model and PDXO maintain the histopathological features of the primary tumors. **A** Microscopy images depicting major morphological phenotypes of PDXOs. **B** Representative images of H&E staining and immunohistochemistry for ER, PR, and HER2 in PDXOs and the tumor tissues of PDX mice. PDXO, PDX-derived organoid; H&E, hematoxylin & eosin; ER, estrogen receptor; PR, progesterone receptor; HER2, human epithelial receptor 2

**Fig. 4:**
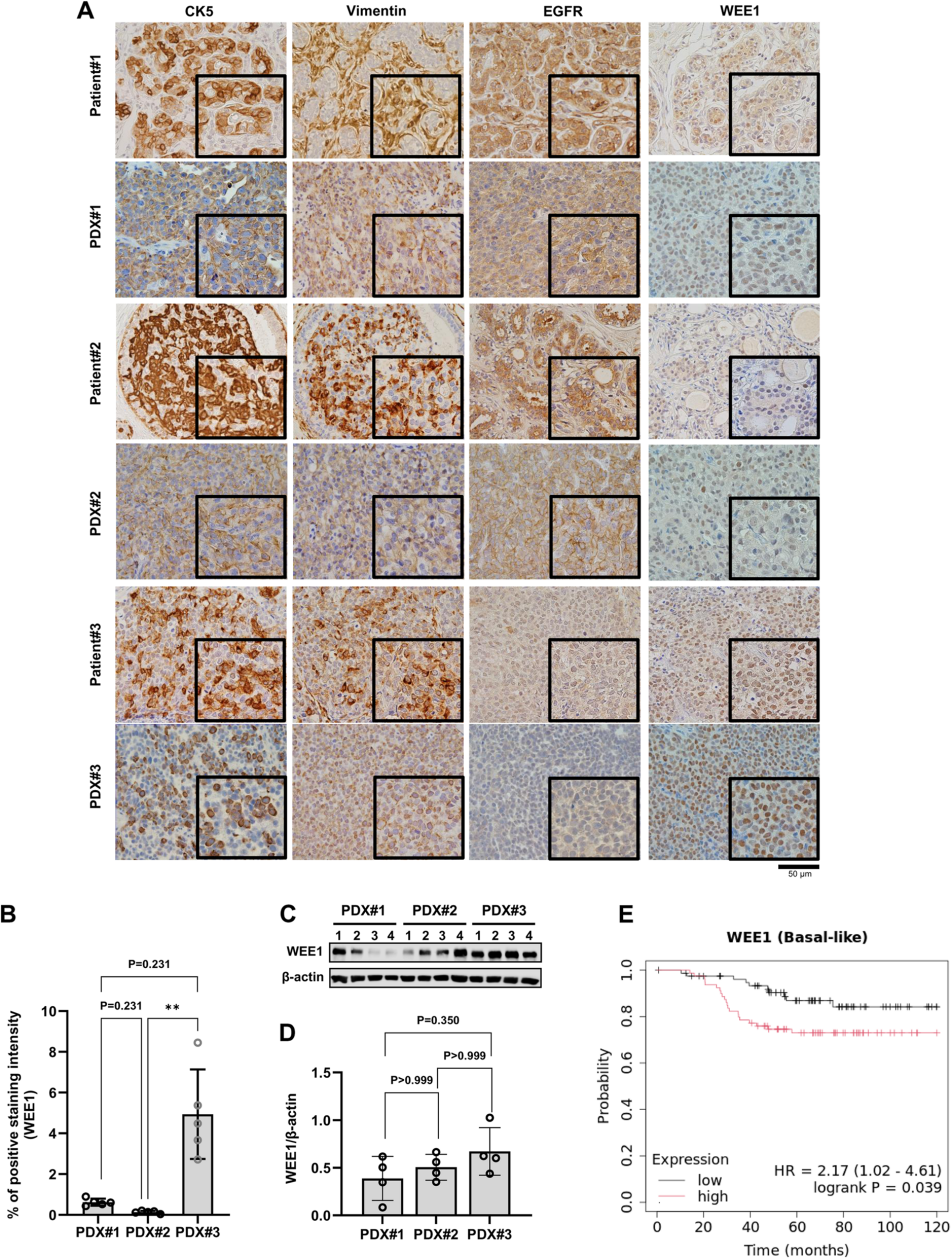
High WEE1 expression was associated with poor survival outcomes in TNBC patients with basal-like subtype. **A** Representative immunohistochemistry images of CK5, vimentin, EGFR, and WEE1 in patients and PDX tumor tissues. **B** The quantitative analysis of immunohistochemistry of PDX tumor tissues. **C**, **D** Western blot image and densitometry band quantification (*n* = 4) of WEE1 normalized by ß-actin in PDX tumor tissues. **E** Overall survival analysis in basal-like patients (*n* = 158) with a mean follow-up of 120 months defined by PAM50 and IHC using the KM-Plotter database. All data were presented as mean ± standard deviation. *P < 0.05. **P < 0.01 using One-way ANOVA followed by Tukey's multiple comparison test. CK5, cytokeratin 5; EGF, epidermal growth factor receptor; PDO, patient-derived organoid; PDX, patient-derived xenograft; PDXO, PDX-derived organoid; PAM50, prediction analysis of microarray 50; KM-plotter, Kaplan–Meier Plotter

We also successfully established PDXO#1, #2, and #3 from fresh PDX tumors by subculturing up to five times. PDXOs displayed a more compact structure than PDOs and grew as solid spheres of varying diameters (Fig. 3a). Immunohistochemistry results showed that all PDXs and PDXOs matched ER-, PR-, and HER2-negative status of the original TNBC patients (Fig. 3b). CK5 and vimentin were strongly expressed in tumor cells within the PDX#1, #2, and #3 tumor tissues, while EGFR was highly expressed in PDX#1 and #2 tumor tissues, but not in PDX#3, consistent with IHC results from patient tissues (Fig. 4a). WEE1, a cell cycle regulator in the G2/M and S phases [20], was predominantly detected in the nuclei of tumor cells within PDX#1, #2, and #3 tumor tissues and was highly expressed in PDX#3 tumor tissue compared to the other tumor tissues (Fig. 4a and b). Likewise, the Western blot analysis revealed that PDX#3 tumors exhibited higher WEE1 expression than the other PDX tumors (Fig. 4c and d).

### High WEE1 expression was associated with poor survival outcomes in patients with TNBC with the BL subtype

TNBC with basal-like (BL) subtypes were identified by immunohistochemical markers, including cytokeratins (CK5, CK6, CK14, or CK17), vimentin, and EGFR [2, 21–24]. IHC revealed high expression of CK5 and vimentin in tumors of patients #1, #2, and #3, whereas EGFR was abundantly expressed in tumors from patients #1 and #2 but not in patient#3 (Fig. 4a). WEE1 was primarily localized in the nuclei of tumor cells and was more abundant in the tumor from patient#3 than in the other patients (Fig. 4a).

To assess the prognostic value of WEE1 expression in patients with TNBC, we analyzed the association between WEE1 mRNA expression levels and OS using an online KM-Plotter database in patients with TNBC (*n* = 158) with a mean follow-up of 120 months, stratified by PAM50 and IHC. High WEE1 gene expression significantly correlated with short OS in patients with TNBC-BL (HR = 2.17, 95% CI 1.02 − 4.61, *p* = 0.039, Fig. 4e). We also stratified the KM-Plotter database based on the Lehmann subtype to assess the relationship between WEE1 expression and outcomes in patients with non-TNBC-BL [2]. Although statistical significance was not reached due to the limited cohort size, only BL1-type patients (*n* = 44) with elevated WEE1 expressions had shorter OS. (HR = 1.67, 95% CI 0.47 − 5.92, *p* = 0.42) (Supplementary Fig. 1).

### WEE1 inhibitor AZD1775 suppressed tumor growth in TNBC-PDX mice

**Fig. 5:**
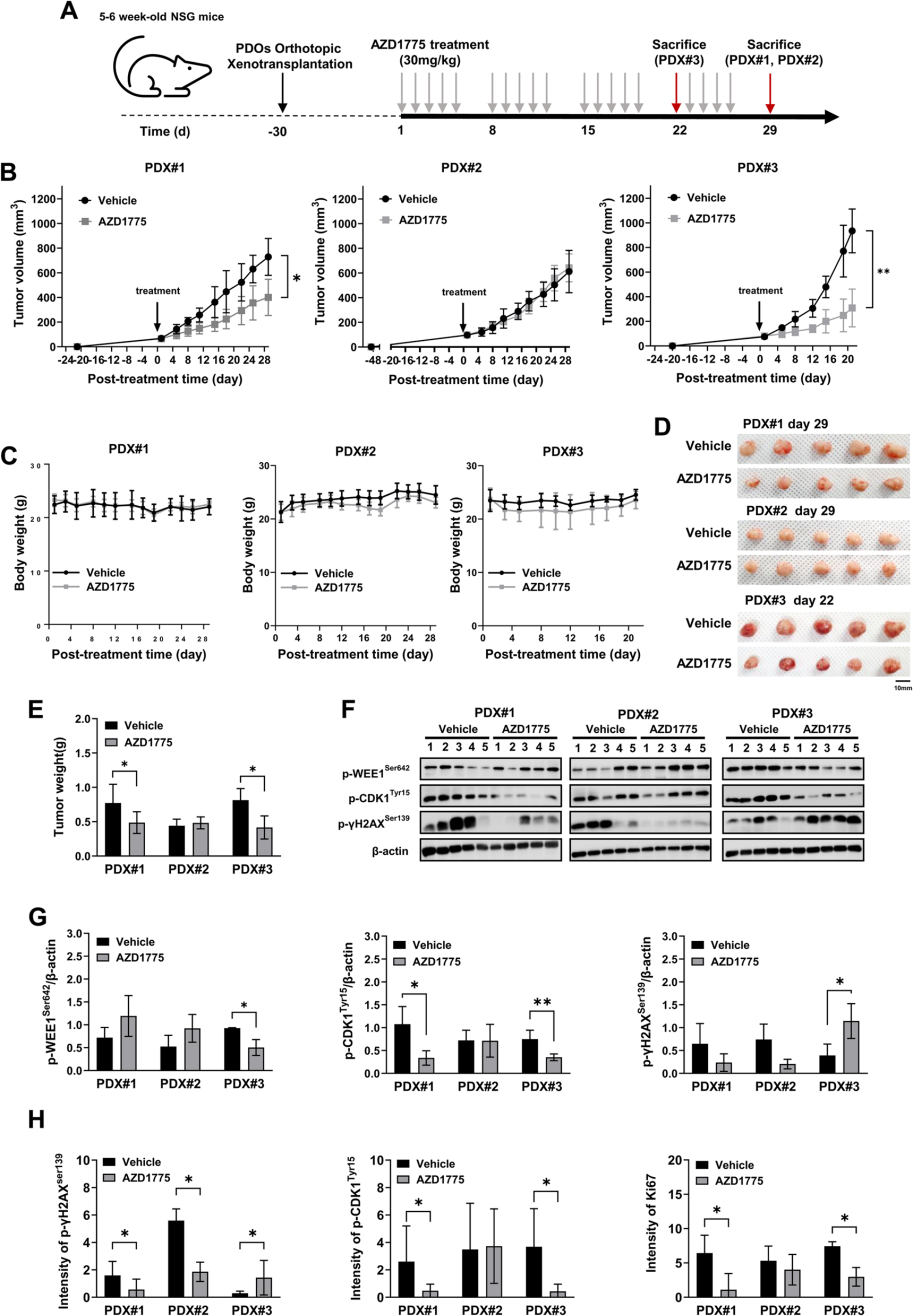
Oral administration of AZD1775 suppressed tumor growth by increasing γH2AX phosphorylation while decreasing CDK1 phosphorylation and Ki67 expression in PDX mice. **A** Experimental design and timeline. **B**, **C** Tumor volume and body weights measured two times weekly in PDX mice treated with vehicle, or AZD1775 (30 mg/kg daily oral gavage) (*n* = 5). **D**, **E** Gross images and wet weights (*n* = 5) of PDX tumors. **F**, **G** Western blot images and densitometry band quantification (*n* = 5) of p-WEE1, p-CDK1, and p-γH2AX normalized by ß-actin in PDX tumors. **H** Quantitative analysis (*n* = 5) of immunohistochemistry of Ki67, p-CDK1, and p-γH2AX in tumors. All data were presented as mean ± standard deviation. *P < 0.05. **P < 0.01. unpaired t-test. PDX, patient-derived xenograft; CDK1, cyclin-dependent kinase 1

Next, we evaluated the WEE1-targeted therapeutic potential in TNBC-PDX models. Figure 5a illustrates the timeline of the in vivo study examining the anti-cancer effect of AZD1775 in PDX#1, #2, and #3 mice. PDX mice (#1[P4], #2[P4], and #3[P3]) bearing tumors larger than 50 mm^3^ were treated with 30 mg/kg of AZD1775 via oral gavage for 3–4 weeks, with 5 days on and 2 days off. Compared to controls, AZD1775 administration significantly inhibited tumor growth in PDX#1 (*p* = 0.01587) and PDX#3 mice (*p* = 0.00794) but not in PDX#2 mice (*p* = 0.46340) (Fig. 5b). No significant change in body weight was observed in any animal following AZD1775 treatment (Fig. 5c). All tumors were excised from PDX mice when the control tumor volume reached 800 mm^3^ (Fig. 5b and d). AZD1775 treatment significantly reduced the wet weights of excised tumors in PDX#1 mice (control vs AZD1775; 0.772 ± 0.272 g vs 0.486 ± 0.158 g, *p* = 0.0476) and PDX#3 mice (control vs AZD1775; 0.814 ± 0.168 g vs 0.416 ± 0.168 g, *p* = 0.0159) compared to controls (Fig. 5e).

### AZD1775 suppressed tumor growth and was associated with increased γH2AX phosphorylation while decreasing CDK1 phosphorylation and Ki67 expression

Phosphorylated cyclin-dependent kinase 1 (CDK1), which is involved in unrestricted cell proliferation is proposed as a promising target for cancer treatment [25]. Phosphorylated γH2AX (p-γH2AX) indicates DNA damage caused by replication stalling [26, 27]. To investigate the anti-tumor mechanisms of AZD1775, we examined the protein expression levels of p-WEE1 (Ser642), p-CDK1 (Tyr15), and p-γH2AX (Ser139) in PDX tumor lysates using Western blot (Fig. 5f). Compared to controls, AZD1775 treatment resulted in a significant decrease in p-CDK1 (Tyr15) levels in PDX#1 (control vs AZD1775; 1.159 ± 0.282 vs 0.343 ± 0.208, *p* = 0.0159) and PDX#3 (control vs AZD1775; 0.977 ± 0.269 and 0.645 ± 0.111, *p* = 0.00794) (Fig. 5g). Only PDX#3 treated with AZD1775 had significantly lower p-WEE1 levels (control vs AZD1775; 1.067 ± 0.252 and 0.813 ± 0.370, *p* = 0.0158) and higher p-γH2AX levels (control vs AZD1775; 0.391 ± 0.249 and 1.144 ± 0.380, *p* = 0.0317) (Fig. 5g).

IHC images, consistent with Western blot results, revealed that AZD1775 treatment significantly decreased p-CDK1 (Tyr15) expression in PDX#1 and #3 while increasing p-γH2AX (Ser139) expression in PDX#3 (*p* < 0.05, Fig. 5h, and Supplementary Fig. 2). Ki67, a widely used proliferation marker for human tumor cell proliferation [28], decreased in PDX#1 and #3 tumors treated with AZD1775 (*p* < 0.05, Fig. 5h, and Supplementary Fig. 2).

### AZD1775 increased the population of G2/M phase cells and p-γH2AX-positive cells, resulting in aneuploidy, DNA damage, and cell death in PDXOs

**Fig. 6:**
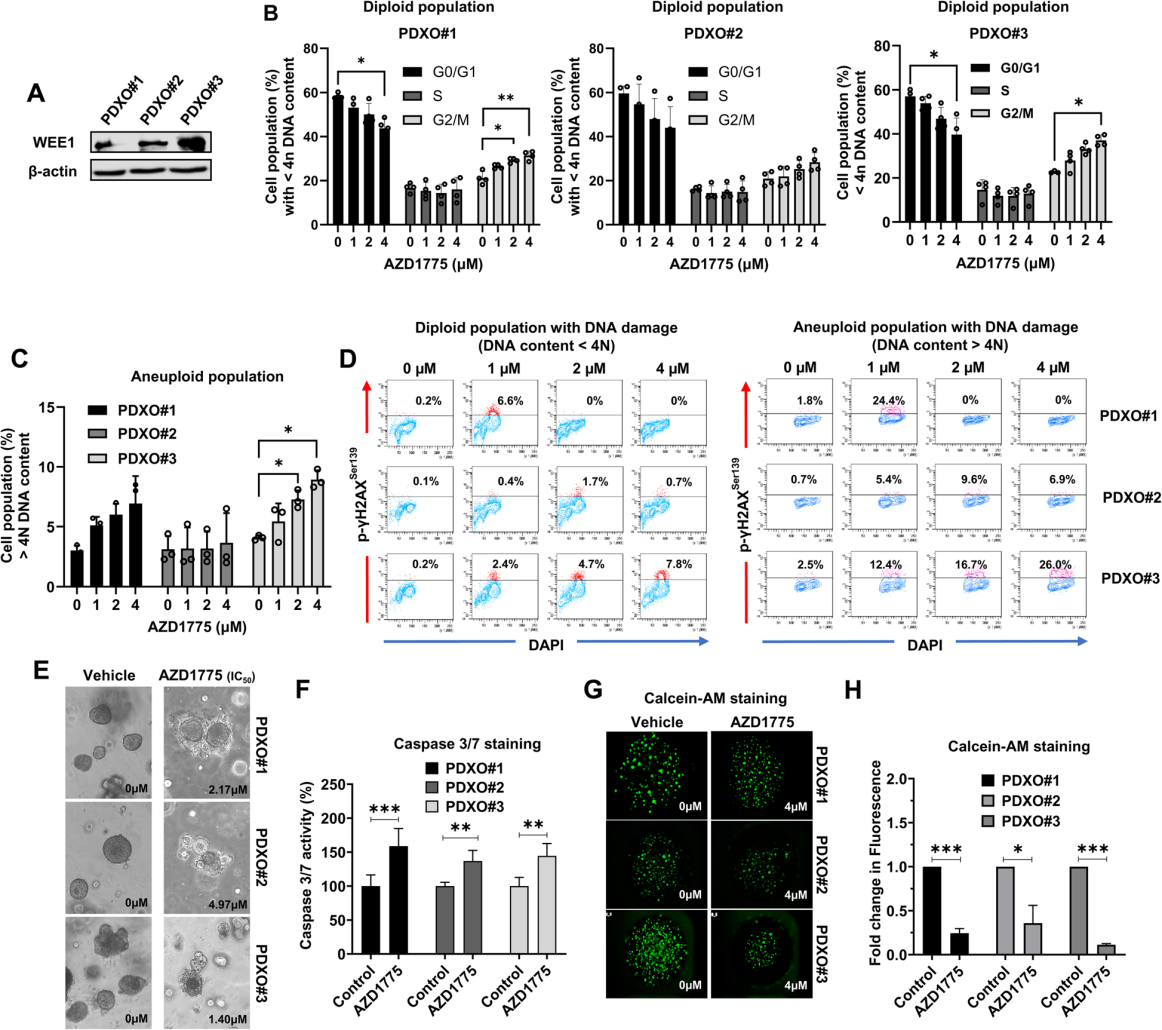
AZD1775 led to abnormal G2/M arrest and increased p-γH2AX-positive aneuploid population, leading to aneuploidy, DNA damage, and cell death in PDXOs. **A** Western blot images of WEE1 in PDXOs. **B**, **C** Propidium iodide-based cell cycle analysis of cells with diploid and aneuploid DNA contents in PDXOs treated with AZD1775 (1, 2, and 4 µM) for 24 h using flow cytometry (*n* = 4). **D** p-γH2AX detection in cells with diploid and aneuploid DNA contents in PDXOs treated with AZD1775 (1, 2, and 4 µM) for 48 h using flow cytometry. **E** Microscopy images of PDXOs treated with AZD1775 (IC_50_) for 7 days. **F** The activity of Caspase 3/7 staining after 7 h exposure to IC_50_ concentration of AZD1775 (*n* = 3) (**G**, **H**) Representative images of PDXOs stained with Calcein-AM and Calcein fluorescence intensity (*n* = 3) in PDXOs treated with 4 μM AZD1775 for 6 days. All data were presented as mean ± standard deviation. *P < 0.05. **P < 0.01. ***P < 0.001 using unpaired t-test. PDX, patient-derived xenograft; PDXO, PDX-derived organoid; Calcein-AM; calcein- acetoxymethyl-ester

Consistent with WEE1 results from patients and PDX tumor tissues, a representative Western blot showed higher WEE1 expression in PDXO#3 than in PDXO#1 and #2 (Fig. 6a). PDXOs were cultured as 2D monolayers in a collagen-type-I-coated and treated with various concentrations of AZD1775 for 24 h to investigate the DNA content and cell cycle in PDXOs treated with AZD1775. Cell cycle analysis by flow cytometry, based on DNA content (diploid) measurement using PI staining, showed that AZD1775 treatment significantly increased the proportion of cells in the G2/M phase and decreased the proportion of cells in the G0/G1 phase in a dose-dependent manner in PDXO#1 and #3 (Fig. 6b). Following AZD1775 treatment, PI staining showed a significant dose-dependent increase in cells with ˃ 4 N DNA contents (aneuploidy) in PDXO#3 (Fig. 6c).

Next, we examined cell populations with aneuploid and DNA damage by flow cytometry based on double staining with p-γH2AX and DAPI in PDXOs treated with AZD1775. As expected, the flow cytometric analysis revealed that the proportions of p-γH2AX-positive cells with aneuploidy (> 4 N DNA contents) analyzed by DAPI were higher in PDXO#1 and #3 than in PDXO#2 (Fig. 6d), indicating AZD1775 led to the severe DNA damage and aneuploidy in PDXO#1 and #3.

In the IC50 of AZD1775 using the HTS technique, PDXO#3 (1.40 µM) showed the highest sensitivity compared to PDXO#1 (2.17 µM) and PDXO#2 (4.97 µM) (Table 3 and Fig. 6e). Caspase 3/7-stained apoptotic cells also significantly increased in all PDXOs treated with AZD1775 at the IC50 (Fig. 6f). As shown in representative fluorescent images and quantitative analysis of calcein-AM staining in PDXOs treated with AZD1775 (4 µM) for 3 days, PDXO #3 showed the greatest decrease in casein-AM-positive healthy cells (green fluorescence) (Fig. 6g and h).

**Table 3:** IC_50_ (µM) values of anti-cancer drugs in PDOs and PDXOs assessed using CellTiter-Glo® assay

Target	Drugs	PDXO#1 (µM)	PDXO#2 (µM)	PDXO#3 (µM)	PDO#3 (µM)	PDO#4 (µM)	PDO#5 (µM)
DNA synthesis	Doxorubicin	0.12	0.04	0.13	0.13	0.01	1.47
Microtubule detachment	Paclitaxel	200 ↑	12.99	0.13	0.05	0.08	0.18
Microtubular depolymerization	Docetaxel	51.5	4.20	0.18	0.06	0.22	0.01
Microtubule function	Eribulin	0.01	3 ↑	0.61	0.66	0.01	0.005
Thymidylate synthase	Capecitabine	200 ↑	200 ↑	200 ↑	200 ↑	200 ↑	200 ↑
DNA synthesis	Gemcitabine	1.03	0.27	0.47	0.69	0.1	0.19
DNA synthesis	Carboplatin	100 ↑	66.49	96.89	41.03	50.73	15
DNA synthesis	Cisplatin	100 ↑	100 ↑	42.89	100 ↑	46.85	100 ↑
CDK4/6	Palbociclib	0.88	4.73	9.47	12.16	1.86	10.73
EGFR, ErbB2,Erk-1/2, AKT, CyclinD	Lapatinib	0.05	0.14	0.67	3.08	0.22	6.25
PARP	Olaparib	50 ↑	50 ↑	39.64	50 ↑	42.33	48.69
PIK3CA	Taselisib	0.03	0.04	2.03	3 ↑	0.1	1.15
mTOR	Everolimus	0.03	3.9	0.47	0.69	7.1	14.2
ATR	AZD6785	5.14	9.42	10 ↑	10 ↑	3	2.78
WEE1	AZD1775	2.17	4.97	1.40	0.86	1.8	0.19
HDAC	Quisinostat	0.01	0.01	0.27	0.04	0.01	0.01
Tankyrase	XAV-939	3 ↑	3 ↑	2.58	1.60	3 ↑	3 ↑
A small-Molecule WNT	Wnt-C59	2.73	3 ↑	3 ↑	3 ↑	3 ↑	3 ↑

*PDO* patient-derived organoid, *PDXO* PDX-derived organoid

### PDOs and PDXOs had a strong concordance in their responses to anti-cancer drugs

**Fig. 7:**
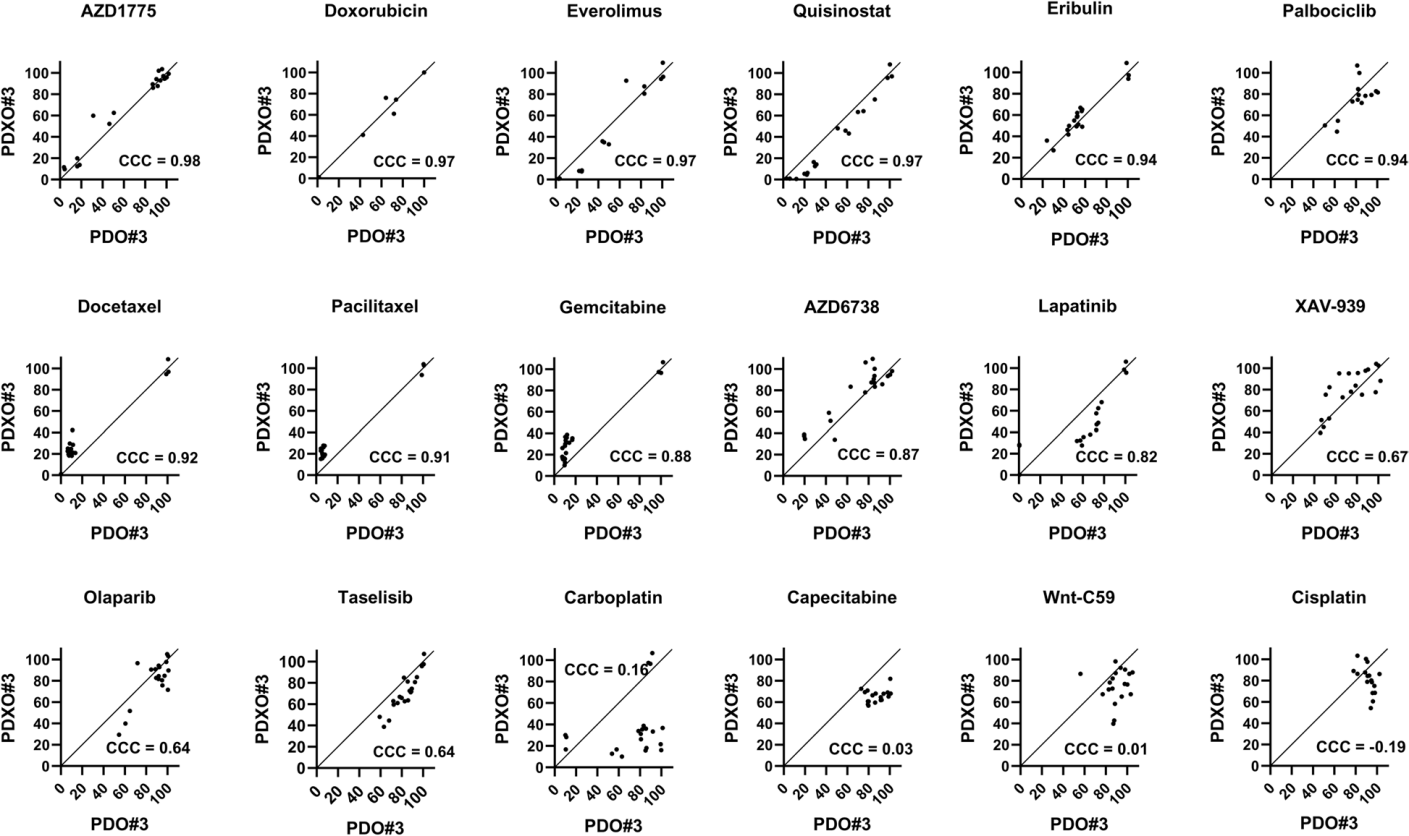
PDOs and -PDXOs had a high concordance in their responses to anti-cancer medicines, including AZD1775. Scatter plots (correlation) of cell viability (%) against anti-cancer drugs at different concentrations in PDOs (*n* = 3) and PDXOs (*n* = 3). Perfect correlation, CCC > 0.99; Substantial correlation, 0.95 to 0.99; moderate correlation, 0.90 to 0.95; poor correlation, < 0.90. PDO, patient-derived organoid; PDXO, PDX-derived organoid; CCC; Lin’s Concordance Correlation Coefficient

To assess the concordance of drug responses between PDOs and PDXOs, 3D cell viability was evaluated in a dose-dependent manner using the CellTiter-Glo® assay in an HTS system. The correlation strength between PDO#3 and PDXO#3 in response to anti-cancer drugs was determined using Lin’s Concordance Correlation Coefficient (CCC), as described by L.I. Lin [29]. CCC values were interpreted as follows: < 0.90, poor; 0.90 − 0.95, moderate; 0.95 − 0.99, substantial; and > 0.99, almost perfect correlation [30]. The correlation coefficients between PDO#3 and PDXO#3 treated with four anti-cancer drugs (AZD1775, CCC = 0.98; doxorubicin, CCC = 0.97; everolimus, CCC = 0.97; quisinostat, CCC = 0.97), which induced dose-dependent cytotoxicity, indicated a substantial correlation (Fig. 7). A moderate correlation was observed for eribulin (CCC = 0.94), palbociclib (CCC = 0.94), docetaxel (CCC = 0.92), and paclitaxel (CCC = 0.91) (Fig. 7). In contrast, poor correlation values were recorded for gemcitabine (CCC = 0.88), AZD6738 (CCC = 0.87), lapatinib (CCC = 0.82), olaparib (CCC = 0.64), taselisib (CCC = 0.64), carboplatin (CCC = 0.16), capecitabine (CCC = 0.03), and Wnt-C59 (CCC = 0.01).

### PDOs and PDXOs may serve as surrogates for screening initial drug response and selecting sensitive drugs for personalized therapy of TNBC patients

IC50 values were used to determine drug effectiveness (sensitivity). The therapeutic efficacy of 18 anti-cancer drugs in PDOs and PDXOs was evaluated based on IC50 values calculated using CellTiter-Glo® Luminescent Cell Viability Assay in an HTS system. As shown in Table 3, anti-cancer drug responses varied among individual PDOs and PDXOs.

**Fig. 8:**
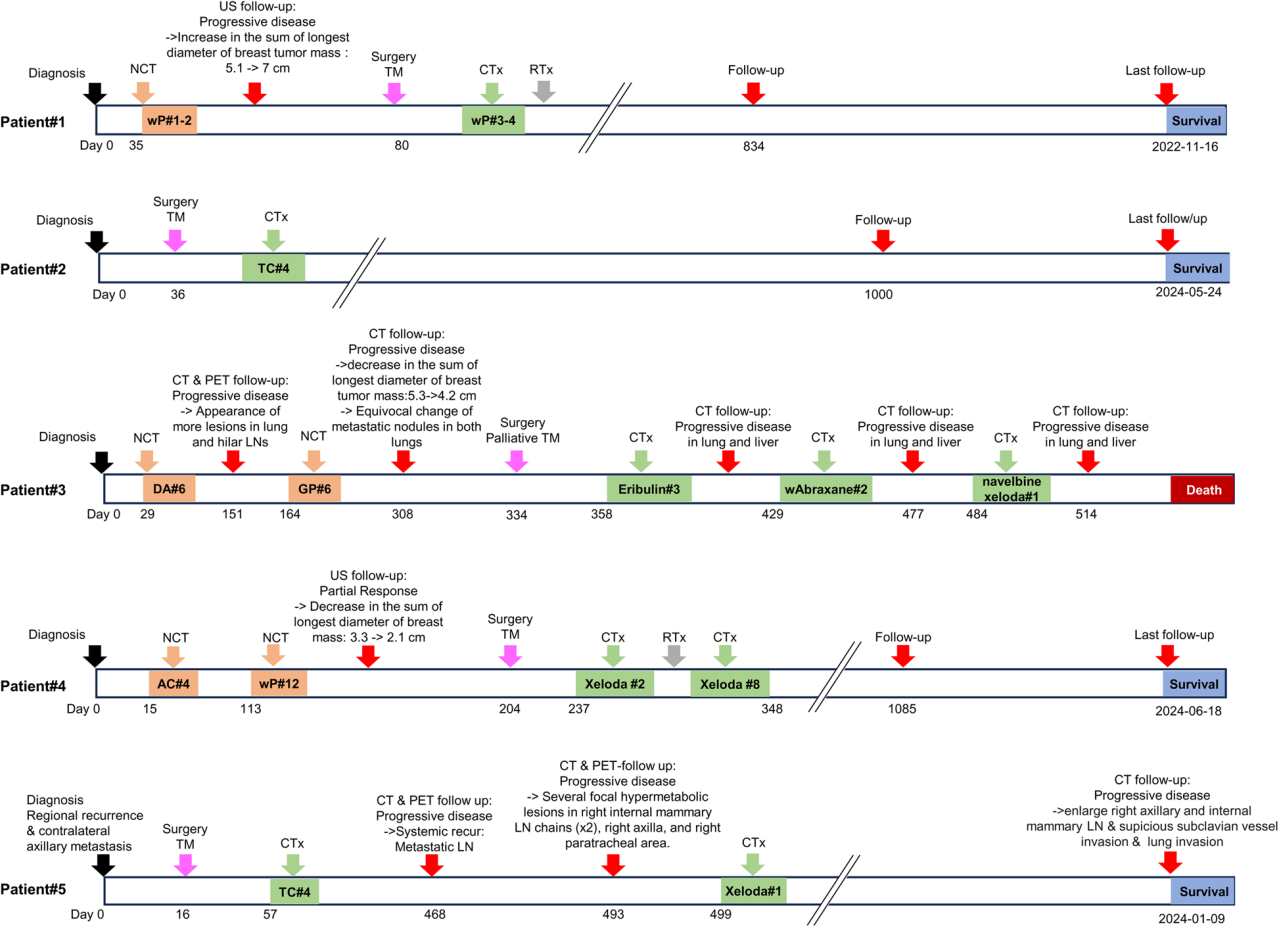
Timeline of the personal information, including details such as NCT, CTx, and follow-up documentation of residual disease, its spread, or additional malignancies. NCT, neoadjuvant chemotherapy; CTx, adjuvant chemotherapy; RTx, radiotherapy; TM, total mastectomy; CT, computed tomography; PET, positron emission tomography; AC, doxorubicin + cyclophosphamide; TC, docetaxel + cyclophosphamide; wP, weekly paclitaxel; DA, docetaxel + Adriamycin; GP, gemcitabine + paclitaxel; w, week

Drug responses in PDO and PDXO were compared with patients’ responses to neoadjuvant chemotherapy (NCT) or conventional chemotherapy (CTx) based on clinical records (Table 3 and Fig. 8). Patient#1 initially received weekly paclitaxel (wP) as part of NCT, but treatment was discontinued due to tumor growth. Subsequently, the patient underwent a total mastectomy and resumed chemotherapy. Following this treatment regimen, the patient survived without recurrence on a 3-year follow-up. After the initial response to NCT, the breast tumor mass increased in size (5.1 cm to > 7.0 cm) (Fig. 8). Consistent with the patient’s tumor response, PDXO#1 did not respond to paclitaxel at 200 µM (Table 3).

Patient#2 received docetaxel + cyclophosphamide (TC) as part of CTx after undergoing a total mastectomy and survived without recurrence during a 3-year follow-up (Fig. 8). Although TC treatment was not directly tested in the models, PDXO#2 displayed a 50% cytotoxic response to docetaxel at 4.2 µM (Table 3).

Patient#3 was directed to palliative care due to initial M1-stage disease. After initially receiving palliative CTx, including docetaxel + Adriamycin (doxorubicin) (DA) and gemcitabine + paclitaxel (GP), the breast tumor mass decreased in the sum of the longest diameter (5.3 to > 4.2 cm). (Fig. 8). Although combination treatments were not directly screened, similar to NCT results in patient#3, both PDO#3 and PDXO#3 showed a 50% cytotoxic response to doxorubicin (0.13 µM), docetaxel (0.18 − 0.06 µM), gemcitabine (0.47 − 0.69 µM), and paclitaxel (0.05 − 0.13 µM) (Table 3). These drugs also exhibited CCC values ranging from moderate to substantial, except for gemcitabine (CCC = 0.88) (Fig. 7). Subsequently, patient#3 underwent a palliative mastectomy followed by CTx, including eribulin, nab-paclitaxel (Abraxane), vinorelbine (Navelbine), and capecitabine (Xeloda), but later developed progressive lung and liver metastases and eventually died (Fig. 8). In contrast to the patient’s clinical outcome, PDO#3 and PDXO#3 exhibited responses to these drugs (Table 3), as these models were derived from primary tumor tissue, which did not originate from metastatic regions.

Patient#4 received NCT with doxorubicin + cyclophosphamide (AC) and paclitaxel, followed by a total mastectomy and adjuvant CTx with capecitabine (Xeloda), and survived (Fig. 8). The breast tumor mass partially responded to NCT (sum of the longest diameter: 3.3 cm to > 2.1 cm) (Fig. 8). Consistent with NCT findings, PDO#4 showed a 50% cytotoxic response to paclitaxel at 0.08 µM (Table 3).

Patient#5 underwent a right lumpectomy and sentinel lymph node biopsy followed by radiotherapy 10 years prior but later developed local recurrences in the breast, axillary intramammary lymph nodes (LNs), and right supraclavicular LNs (Fig. 8). Despite undergoing total mastectomy and CTx with TC and capecitabine (Xeloda), the patient experienced progressive metastases involving the right internal mammary LN chains, right axilla, and right paratracheal region (Fig. 8). Although TC treatment was not tested in vitro, PDO#5 exhibited 50% cytotoxicity in response to docetaxel at 0.01 µM (Table 3). However, when evaluated for TC and capecitabine (Xeloda), used in CTx, PDO#5 showed no cytotoxic response to capecitabine at 200 uM (Table 3).

## Discussion

Here, we reported the successful creation of five PDO lines, three PDX mice, and three PDXO lines that retained the immunohistological features of patient’s tumors diagnosed with TNBC. Using the KM-Plotter database, we found that high WEE1 expression in TNBC and BL subtype tumors was associated with poor prognosis. We also observed that PDXs and PDXOs generated from patients with TNBC, particularly those with the BL subtype, which highly expressed WEE1, were very sensitive to the WEE1 inhibitor AZD1775. This sensitivity was demonstrated through decreased CDK1 phosphorylation, a reduction in G2/M arrest, an accumulation of p-γH2AX, and a reduction in Ki67 expression, suggesting that the WEE1 inhibitor may be a promising therapeutic regimen for these patients. The responses of PDOs and PDXOs to AZD1775 matched those of the respective PDXs. Moreover, HTS analysis revealed a high concordance of anti-cancer drug responses, including AZD1775, between matched PDOs and PDXOs. This finding suggests that PDXOs and PDOs are useful in vitro models for cost-effectively assessing anti-cancer drug efficacy and selecting sensitive anti-cancer drugs in the initial screening for personalized treatment for patients with TNBC.

TNBC is an aggressive and heterogeneous disease that responds poorly to conventional chemotherapy regimens and has limited treatment options [31]. Additionally, TNBC patients with a higher residual cancer burden (RCB) index, a prognostic score [32], exhibit worse relapse-free survival (RFS) compared to those with the hormone-positive breast cancer subtype [32]. Given the limited response to standard therapy and the poor prognosis for TNBC patients, an appropriate preclinical model system for selecting effective anti-cancer drugs and predicting treatment responses is essential. PDOs and PDXs could be valuable models for predicting patients’ responses to anti-cancer drugs and discovering personalized therapeutic strategies [6–12, 33–35]. We produced three PDO, PDX, and PDXO lines that preserved the major immunohistological features of TNBC patients (#1, #2, and #3) with BL subtype highly expressing WEE1, Ki67, CK5, EGFR, and vimentin.

In this study, PDOs from patients with TNBC, particularly those with the BL subtype and high Ki67 expression, generated tumors in PDX mice with lung metastasis, whereas PDO #4 and PDO #5 from patients with TNBC who had low Ki67 expression did not. These findings suggest that tumor cells of patients with TNBC (#1, #2, and #3) are more proliferative and tumorigenic. Recent studies have shown that PDX tumors may underrepresent the intratumoral heterogeneity of patient tumors and accumulate many mutations during PDX establishment, indicating subclonal selection and genomic evolution [33–35]. In our study, following transplantation, PDX tumors grew and metastasized rapidly in the P1 − P4 generations compared to the P0 generation, demonstrating that tumor cells with highly proliferative features were selected during PDX tumor propagation. Our major limitation of the PDX model is the absence of human tumor stroma and the immune system, which play critical roles in tumor growth and progression. To evaluate subclonal reconstruction and genomic evolution in PDO, PDXO, and PDX models, whole-exome sequencing (WES) or targeted gene sequencing should be further studied.

WEE1 has been implicated as an oncogene in multiple cancers [36–38]. In the survival analysis of patients with breast cancer stratified by WEE1 expression in TNBC and non‐TNBC, a significantly shorter metastasis‐free survival was observed in patients with TNBC who had high WEE1 expression but not in those with non-TNBC [18]. The clinical relevance of WEE1 expression has been suggested as a prognostic biomarker in patients with TNBC characterized by high pathological grade, pT2 size, and BL and mesenchymal Lehmann subtypes [2, 18]. Consistent with these reports, patient #3 in our cohort, who had TNBC with the BL subtype and distant metastasis, exhibited high WEE1 expression. In the KM-plotter database, the OS of patients with TNBC and the BL subtype was shorter in the high WEE1 expression group compared to the low WEE1 expression group, suggesting that WEE1 can be used as a biomarker to predict poor prognosis and guide targeted therapy in TNBC with the BL subtype.

TNBC cell lines are more sensitive to WEE1 inhibitors such as AZD1775 and MK‐1775 than non-TNBC cell lines [18]. PDXOs treated with AZD1775 abrogate the G2/M checkpoint by activating CDK1, leading to DNA double-strand breaks as S phase defects and premature mitosis as G2/M defects, resulting in chromosomal aneuploidy and accumulated DNA damage, as evidenced by elevated p-γH2AX level [15, 39–42]. Compared to PDXO#1 and #2 and PDX#1 and #2, PDXO#3 and PDX#3 with substantially high WEE1 expression had the greatest cytotoxicity and tumor growth suppression in response to AZD1775 therapy. This finding demonstrates that TNBC cells with high WEE1 are more sensitive to AZD1775. In AZD1775-treated PDX#1 and #3, we observed an increase in the G2/M phase cell population and aneuploid cells with > 4 N DNA content, as well as a decrease in CDK1 phosphorylation and Ki67 expression consistent with previous results [15, 39–42]. The accumulation of γH2AX phosphorylation, a DNA damage marker, was also observed in the tumor tissues of PDX#3 treated with AZD1775, which was attenuated in PDX#1. We hypothesize that residual cancer cells in PDX#1 tumor, possessing an enhanced DNA repair system against long-term AZD1775 administration, might acquire tolerance, leading to paused cell cycle progression. This hypothesis is supported by the decrease in p-CDK1 and ki67 expression (Fig. 5f-h). WEE1 kinase phosphorylates CDK1 on Tyr15 during the S phase and prevents the S to G2 transition until DNA replication is completed [43], indicating that the reduction of p-CDK1 in PDX#1 treated with AZD1775 resulted from WEE1 inhibition and contributed to an abnormal S/G2 transition. However, premature mitosis was prevented because the DNA damage was repaired, as evidenced by decreased p-rH2AX. Furthermore, the reduction of Ki67, a proliferation marker, in the test group suggested that WEE1 inhibition failed to drive premature mitosis, resulting in a paused cell cycle and tumor growth suppression.

Additionally, the localization of WEE1 in Fig. 4a revealed a significant difference between the cytoplasm and nucleus in patients #1 and #3. The frequency of nuclear WEE1 staining appeared to be preserved from patient #3 to PDX #3, although an increase in nuclear WEE1 expression was observed in PDX #1 compared to patient #1. It has been shown that nuclear WEE1 prevents premature mitotic entry by phosphorylating CDK1 [44]. Therefore, the differences observed between patient #1 and PDX #1 suggest that an elevated functional WEE1 protein is essential for tumor propagation in PDX #1 due to its reliance on the WEE1 function in regulating the cell cycle, which is further supported by the response of PDX #1 to WEE1 inhibition.

These findings provide a scientific rationale for future WEE1-target-driven clinical trials in TNBC patients. However, the clinical relevance of WEE1-targeted therapy must be further assessed in a large scale of TNBC patients with BL subtypes.

Due to patient tissue limitations, generating sufficient PDOs for large-scale in vitro preclinical drug testing is difficult. Bruna et al. demonstrated that drug responses in short-term, 2D-cultured human breast cancer cells derived from PDXs can recapitulate those observed in vivo*.* [45]. However, organoid culture systems through 3D surrounding extracellular matrix (ECM) components with other growth supplements to stimulate cell growth and maintain self-renewal capacity [8] are considered more comprehensive models in oncology, as they accurately recapitulate the histological, and molecular characteristics and clinical response of the original tumors [46]. Therefore, PDXOs, as 3D models, could provide initial drug sensitivity data and help determine the effective treatment for TNBC patients [13]. In our study, PDXOs preserved immunohistology features and the therapeutic drug responses of the corresponding PDOs and PDXs. We produced PDOs and PDXOs that recapitulated major immunohistological features of TNBC patients. One limitation of our study is that only three PDOs (#1, #2, and #3) were engrafted as PDXs; moreover, PDO #1 and #2 were exhausted without evaluating drug response. However, the dose–response and cell viability between PDO#3 and PDXO#3 to anti-cancer drugs with high sensitivity, excluding gemcitabine and palbociclib, showed a good correlation (0.95 < CCC < 0.99), demonstrating that PDXOs can mirror the drug responses of PDOs. Given the high degree of genomic characterization data available, PDXOs could serve as a valuable model for drug development. Low IC50 values indicate potent drug efficacy at low concentrations, reducing systemic toxicity. We measured the IC50 values of 18 anti-cancer drugs in three PDOs and three PDXOs using an HTS system. The IC50 calculated from these PDOs and PDXOs would enable clinicians to prioritize and tailor therapy for corresponding patients.

## Conclusion

**Fig. 9:**
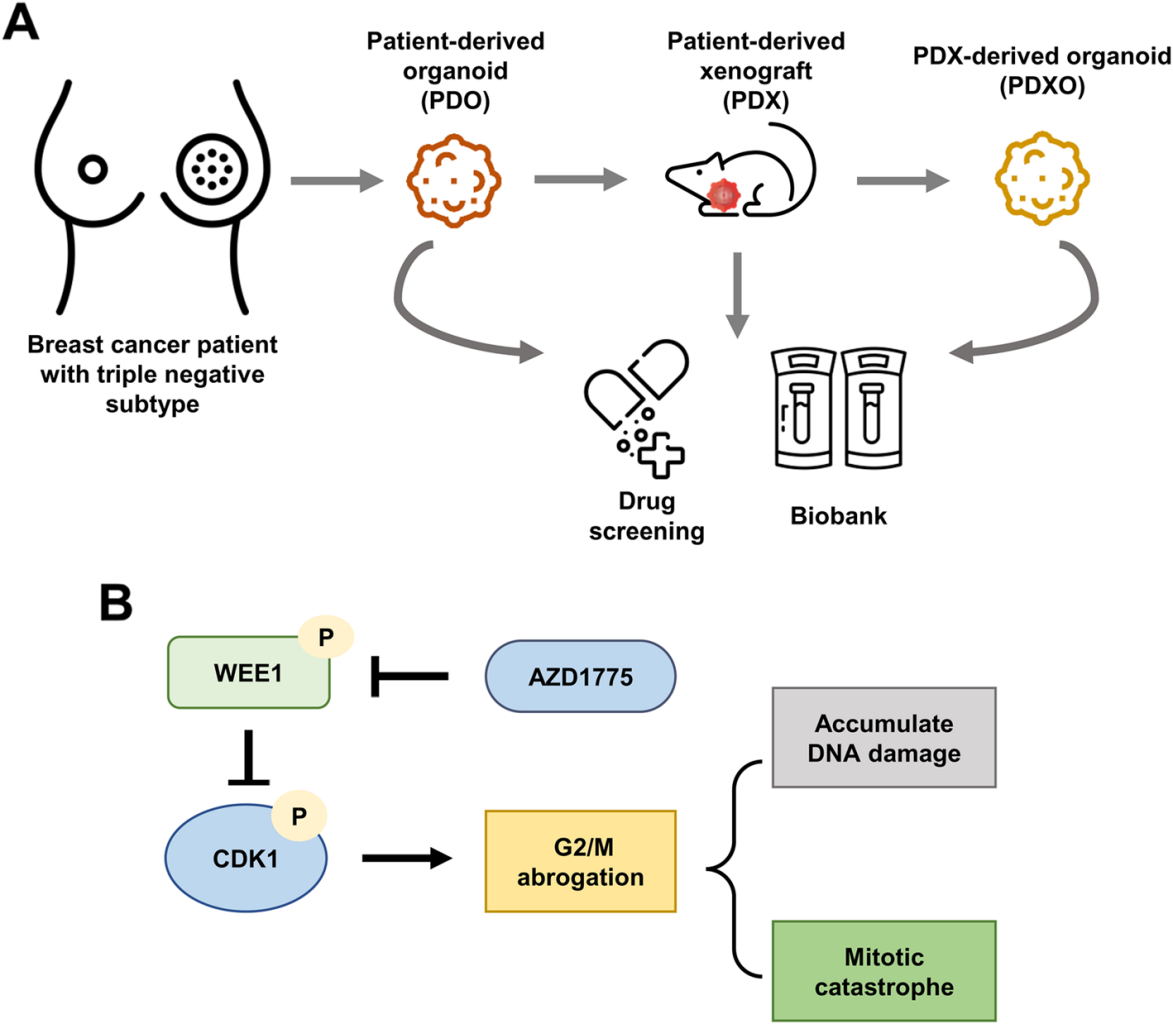
The workflow to establish a biobank with human TNBC-derived organoid lines and perform drug screening including WEE1 targeted therapy. **A** Schematic diagram showing the workflow. Both PDO and PDXO, which retained histological characteristics of human TNBC tumor tissue, are stored in a biobank and treated with anti-cancer drugs. **B** Proposed mechanism of action of the WEE1 inhibitor (AZD1775) in TNBC-derived organoid lines. AZD1775 inhibits the phosphorylation of WEE1, increasing CDK1 activity. This blockade leads to the abrogation of the G2/M checkpoint, which results in the accumulation of DNA damage, and finally triggers a mitotic catastrophe. TNBC; triple-negative breast cancer; PDO, patient-derived organoid; PDXO, PDX-derived organoid; CDK1, cyclin-dependent kinase 1

We successfully established PDOs, PDXOs, and PDX models from surgical tumor tissues of patients with TNBC (Fig. 9a). These models recapitulated the immunohistologic characteristics of TNBC patient tumors. We demonstrated that the therapeutic impact of AZD1775 was associated with the inhibition of p-CDK1, reduction of G2/M arrest, activation of caspase 3/7, upregulation of p-γH2AX, and all of which are linked to DNA damage, mitotic catastrophe, and apoptosis (Fig. 9b). These results, suggest that WEE1-targeted therapy may provide clinically meaningful benefits in patients with TNBC who have high WEE1 expression.

Our results indicate that PDOs and PDXOs can be surrogates to predict anti-cancer drug responsiveness and select sensitive anti-cancer drugs, thereby minimizing drug development costs and time. Additionally, they are expected to be valuable for basic research on carcinogenesis and drug resistance. Therefore, TNBC-PDOs, PDXOs, and PDXs represent valuable tools for predicting drug responsiveness and optimizing treatment strategies for patients with TNBC within a precision medicine strategy.

## Supplementary Information

Supplementary Material 1.

Supplementary Material 2.

## Data Availability

No datasets were generated or analysed during the current study.
