# Supplementary figures and images for: Anti-cancer drug sensitivity testing and preclinical evaluation of the anti-cancer potential of WEE1 inhibitor in triple-negative breast cancer patient-derived organoids and xenograft models
Source: Breast Cancer Res. 2025 Jun 23;27:113. doi: 10.1186/s13058-025-02063-0 (PMC12183918; doi:10.1186/s13058-025-02063-0)

Figure 4

WEE1

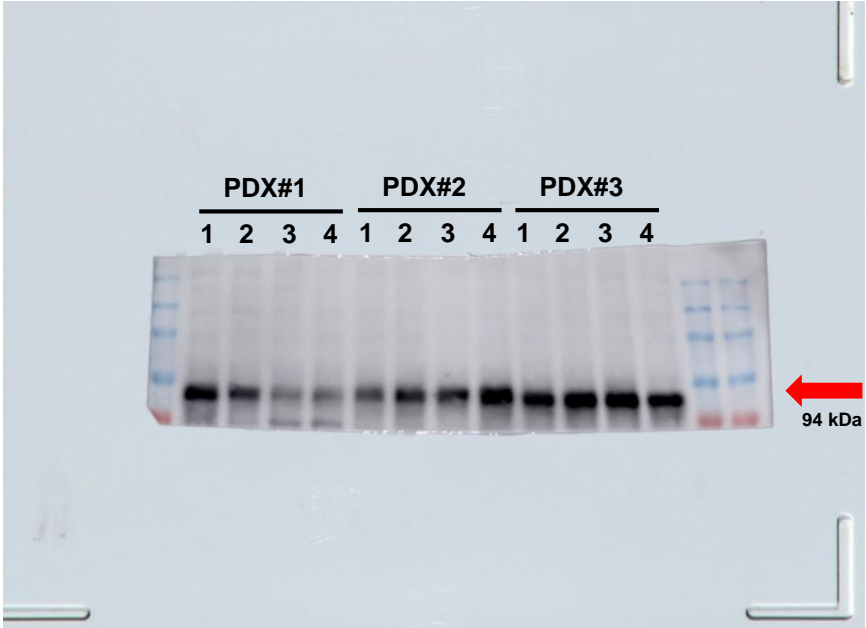

$\beta$ -actin

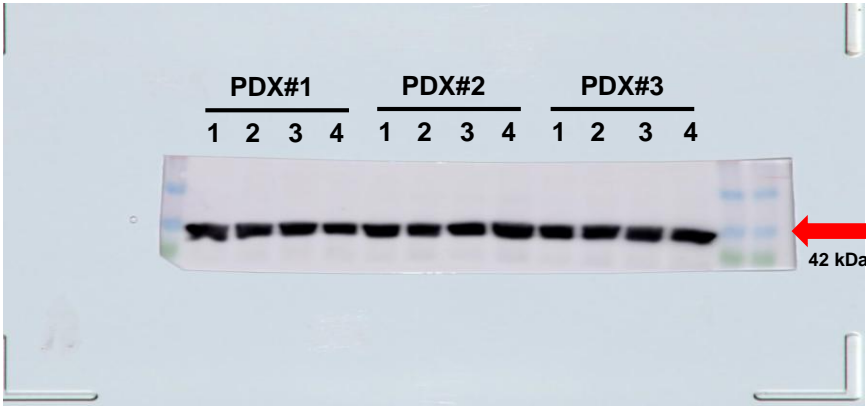

Figure 5

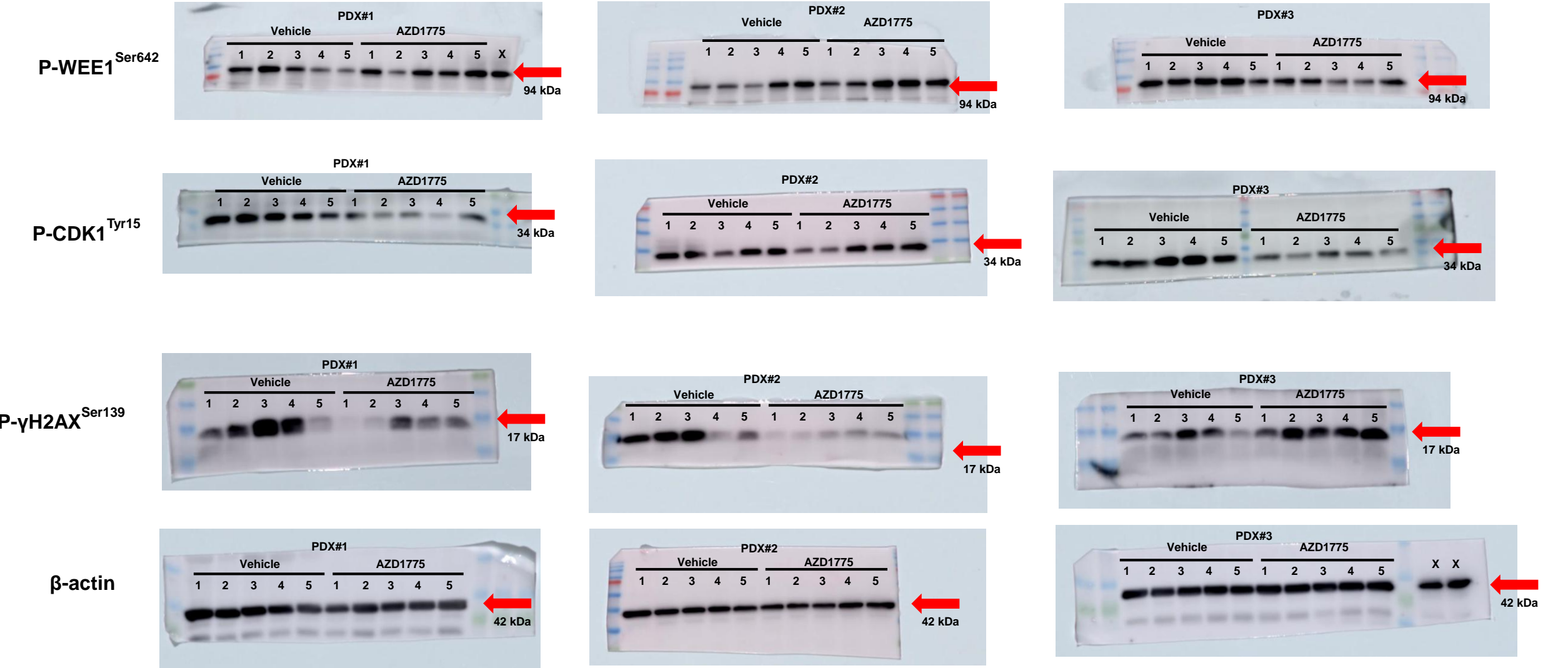

Figure 6

WEE1

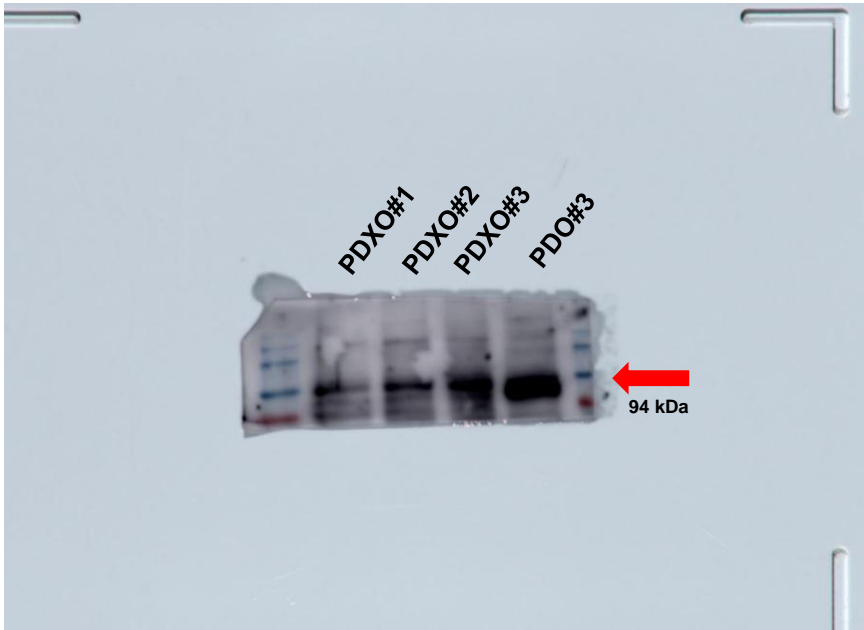

$\beta$ -actin

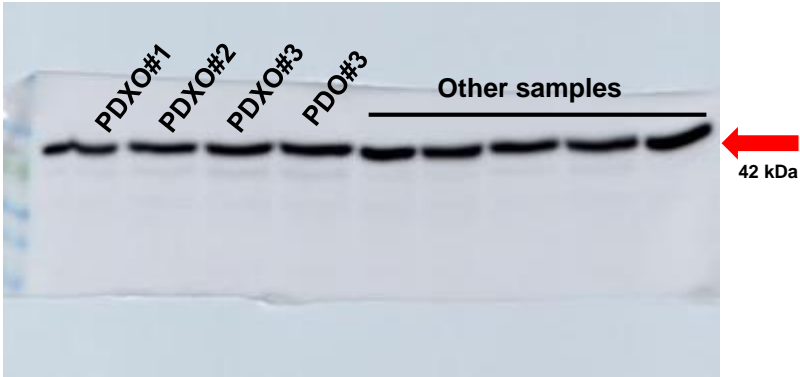

Supplement: Supplementary file 1 — Supplementary Material 1. [file 13058_2025_2063_MOESM1_ESM.pdf]
