# Supplementary material for: Anti-cancer drug sensitivity testing and preclinical evaluation of the anti-cancer potential of WEE1 inhibitor in triple-negative breast cancer patient-derived organoids and xenograft models
Source: Breast Cancer Res. 2025 Jun 23;27:113. doi: 10.1186/s13058-025-02063-0 (PMC12183918; doi:10.1186/s13058-025-02063-0)
Supplement: Supplementary file 2 — Supplementary Material 2. [file 13058_2025_2063_MOESM2_ESM.pdf]

Supplementary Figure 1.

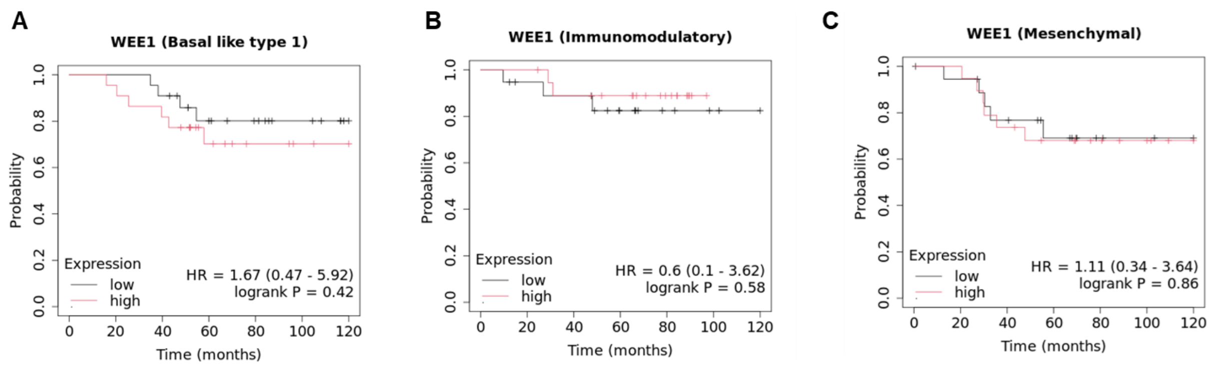

Supplementary Figure 1. Relationship between WEE1 expression and overall survival (OS) analysis over a mean follow-up period of 120 months using the KM-Plotter database.

TNBC subtypes were stratified into basal-like type 1 (A), immunomodulatory (B), and mesenchymal (C) based on the PAM50 and Lehmann classification (HR = 1.67, 95% CI 0.47-5.92, p = 0.42; HR = 0.6, 95% CI 0.1-3.62, p = 0.58; HR = 1.11, 95% CI 0.34-3.64, p = 0.86, respectively).

Supplementary Figure 2.

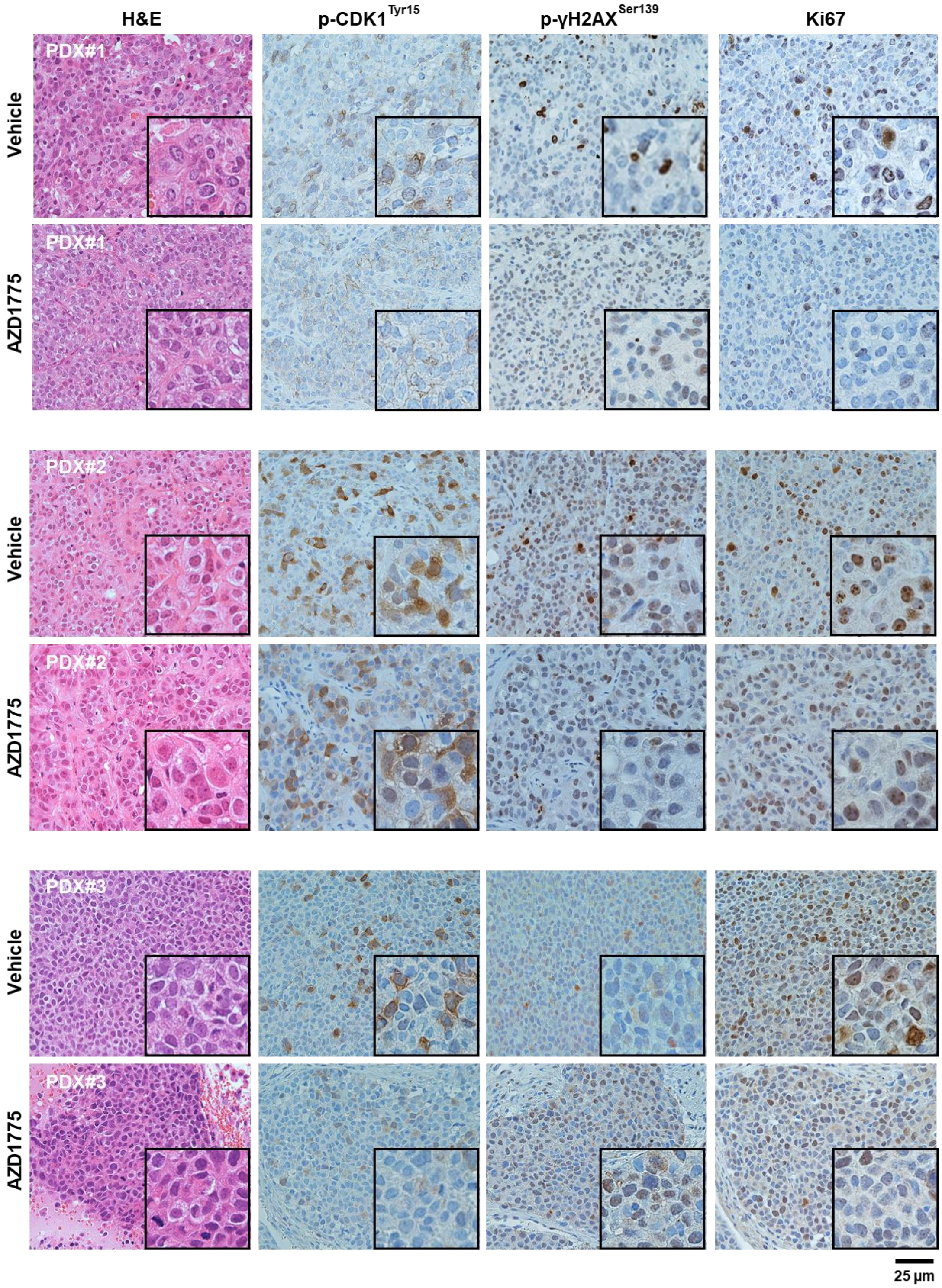

Supplementary Figure 2. Representative images of H&E staining and immunohistochemistry of Ki67, p-CDK1, and p-γH2AX in tumors.
